# Evaluating H_2_ Production by Ultraviolet-Induced Water Splitting over (Cu or Ni)-TiO_2_ Nanoparticle Photocatalysts

**DOI:** 10.1021/acsanm.5c00100

**Published:** 2025-04-17

**Authors:** Meryem Bouchabou, Juan Manuel Rives López, María del Carmen Román Martínez, María Angeles Lillo-Rodenas

**Affiliations:** MCMA Group, Department of Inorganic Chemistry and Materials Institute (IUMA), Faculty of Sciences, 16718 University of Alicante, Ap. 99, E-03080 Alicante, Spain

**Keywords:** photocatalysis, water splitting, hydrogen production, TiO_2_ modification, copper, nickel, thermal reduction, sustainable photocatalysts synthesis

## Abstract

This study explores the surface modification of TiO_2_ P25 with nanosized copper (Cu) or nickel (Ni) for photocatalytic hydrogen production via water splitting. Photocatalysts with 1 or 5 wt % metal loadings were synthesized using a cost-effective impregnation method and were tested as-prepared (XM/P25) and after thermal reduction under hydrogen flow (XM/P25-r). Pre-calcination was omitted to simplify the synthesis. The characterization of the photocataysts revealed key physicochemical, optical, and electronic properties, including the surface distribution and oxidation states of metal species and their interaction with TiO_2_. TEM and EDS showed well-distributed metal species forming nanometric domains on the P25 surface. XPS and Auger spectroscopy allowed the identification of different metal oxidation states and species. After reduction, Cu^0^ and Ni^0^ phases coexisted with oxidized species, enhancing light absorption and reducing the electron–hole recombination, confirmed by UV–vis DRS and PL, respectively. Cu species seemed to exhibit stronger interaction with TiO_2_ than Ni ones. Photochemical results showed that only the thermally reduced photocatalysts led to immediate hydrogen production, highlighting the need for zero-valent metal species for an efficient hydrogen generation. 5Ni/P25-r and 5Cu/P25-r outperformed P25, achieving 331 and 284 μmol H_2_·g_cat_^–1^·h^–1^, respectively, compared to 261 μmol H_2_·g_cat_^–1^·h^–1^ for P25. Although in terms of hydrogen production 5Ni/P25-r slightly outperformed 5Cu/P25-r, the latter demonstrated greater stability, possibly due to the oxidation of Ni during the tests. As-prepared photocatalysts can be activated after extended UV irradiation; for instance, 5Cu/P25 starts producing hydrogen after 10 h of irradiation as a result of the in situ metal reduction, confirmed by the color change in the photocatalyst.

## Graphical abstract

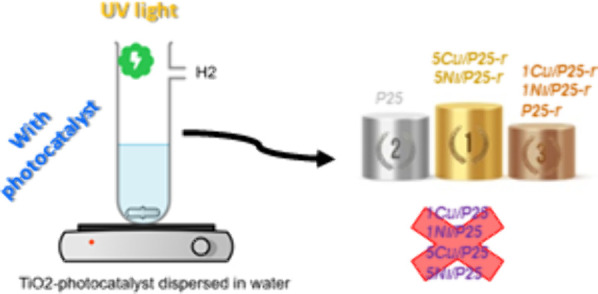

## 1 Introduction

Nowadays, the world faces the dual challenges of reducing carbon emissions and meeting increasing energy demands. In response, hydrogen has emerged as a pivotal element in the energy transition due to its versatility and potential for being produced from renewable sources.^1^

Among the methods to harness the potential of hydrogen, water splitting (WS) stands out as a sustainable and environmentally friendly approach, generating hydrogen using renewable energy sources such as solar power.^2^ A key development in this area was the discovery by Fujishima and Honda that titanium dioxide (TiO_2_) could serve as a photocatalyst to split water into hydrogen and oxygen under ultraviolet light. Since then, TiO_2_ has become widely studied due to its stability, non-toxicity, cost-effectiveness, and potential for large-scale applications. However, its wide band gap (∼3.2 eV for anatase) restricts light absorption to the ultraviolet range, and the rapid recombination of charge carriers hinders its catalytic efficiency.^3^

To address these challenges, extensive research has focused on developing diverse enhancement strategies. These include TiO_2_ modification approaches such as doping, loading it with other elements, forming heterojunctions, and sensitizing TiO_2_ with narrow-band gap semiconductors, among others.^4–8^

The modification of TiO_2_ with metal species via doping, loading or even forming heterojunctions is a growing area of interest due to its potential to significantly enhance photocatalytic performance. These modifications are particularly valuable as they directly influence the electronic properties of TiO_2_, resulting in improved catalytic efficiency under various illumination conditions.

Noble metals such as platinum (Pt) and gold (Au) have shown to greatly enhance the photocatalytic performance of TiO_2_.^9,10^ However, their high cost and scarcity significantly limit their scalability for large-scale hydrogen (H_2_) production. This has driven interest in alternative metals, specifically non-noble metals such as nickel (Ni), copper (Cu), and iron (Fe), which are abundant, cost-effective and, more importantly, exhibit promising catalytic activity when incorporated to TiO_2_-based photocatalysts.^9,11–13^

In this context, focusing on water splitting over TiO_2_-based photocatalysts, Liu et al. reported a hydrogen production rate of 700 μmol g_cat_^–1^ h^–1^ under UV irradiation using 3% Ni-loaded anatase TiO_2_ reduced at 500 °C (3%Ni@gray anatase TiO_2_), whereas unmodified anatase TiO_2_ showed no activity.^14^ Similarly, Zhang et al. demonstrated measurable hydrogen generation with Ni/NiO-TiO_2_ core-shell structures using a xenon lamp, although the performance declined due to photocorrosion of the Ni core.^15^ Kumar et al. observed hydrogen production rates of 198 and 178 μmol g_cat_^–1^ h^–1^ under solar and simulated sunlight, respectively, using bimetallic Cu–Ni–TiO_2_ catalysts.^16^ These catalysts outperformed their monometallic counterparts and, especially, bare TiO_2_, which exhibited much lower rates. Liang et al. further explored dual-single-atom Cu/Co dopants on Li-reduced blue TiO_2_ (Cu–Co SA/BTO) under simulated solar irradiation. Cu SA/BTO achieved a hydrogen generation rate of 881 μmol g_cat_^–1^ h^–1^, which was 7.4 times higher than that of bare BTO.^17^ Co SA/BTO produced 336 μmol g_cat_^–1^ h^–1^, and the Cu–Co SA/BTO catalyst exhibited an impressive hydrogen generation rate of 1238 μmol g_cat_^–1^ h^–1^.^17^ Li et al. highlighted the potential of non-noble metal co-catalysts through their TiO_2_/Co_3_O_4_/Ni catalyst (5% Co_3_O_4_/0.5% Ni), which achieved 123 μmol H_2_ g_cat_^–1^ h^–1^ under UV–visible light over 3 h, an 8.7-fold improvement compared to TiO_2_.^18^ In a novel approach guided by density functional theory (DFT), Jing et al. designed a Cu-loaded and N-doped TiO_2_ (Cu–N–TiO_2_) photocatalyst for efficient solar-driven overall water splitting.^19^ DFT calculations identified monovalent Cu as the optimal co-catalyst for N-TiO_2_, with an ideal Cu mass fraction of around 5% and a discrete surface distribution.^19^ The synthesized catalyst achieved a solar-to-fuel efficiency of 0.2%, producing 1028 μmol H_2_ g_cat_^–1^ h^–1^ under photocatalytic conditions.^19^

The present study focuses on Cu or Ni-modified TiO_2_ photocatalysts as representative examples of non-noble metal-modified materials for hydrogen generation by sacrificial agent-free water splitting. A simple and cost-effective impregnation method was employed to synthesize TiO_2_-based photocatalysts. Commercial TiO_2_ P25 served as the base photocatalyst, with varying Cu or Ni loadings (1 or 5 wt %) to enable a comprehensive analysis to detetermine how these metals and their concentrations influence the photocatalytic activity. The photocatalysts were evaluated under UV light in two forms: in the as-prepared state (denoted as XM/P25, without a calcination step) and after thermal reduction treatment under hydrogen flow (denoted as XM/P25-r), allowing to study the impact of the metal oxidation states on their performance. Hence, this study does not only provide valuable insight into the role of Cu and Ni in enhancing photocatalytic efficiency for hydrogen generation but also investigates the influence of the metal loading and oxidation state, as well as the interaction of each metal with TiO_2_, analyzing if this could induce some change in the oxidation state of the metal. Note that these are factors that can affect the overall photocatalytic process. Another novelty of this work is the analysis of the possible in situ reduction, during the WS test, of the copper species in a representative sample.

## 2 Experimental Section

### 2.1 Preparation of Cu or Ni/TiO_2_ Photocatalysts

The Cu/TiO_2_ or Ni/TiO_2_ photocatalysts were prepared using a simple, cost-effective impregnation method. Specifically, 5 mL of an aqueous solution of Ni­(NO_3_)_2_ or Cu­(NO_3_)_2_ of the concentration required to obtain the desired metal loading, 1 or 5 wt %, was added to 2 g of commercial TiO_2_ powder (Degussa, P25). The mixture was mechanically stirred at room temperature for 2 h, followed by ultrasonication for 30 min. The resulting suspension was dried at 80 °C for 24 h. The dried solids were then ground into a fine powder using a mortar and pestle.

The photocatalysts were tested both as prepared and after being submitted to a thermal reduction treatment, avoiding a calcination step. The thermal reduction treatment was performed under the following conditions: 0.1 g of photocatalyst, 60 mL/min H_2_ flow, a heating rate of 5 °C/min up to 500 °C, and a holding time at 500 °C of 2 h. For comparison, TiO_2_ P25 was also subjected to the same treatment, with the resulting sample denoted as P25-r. The nomenclature for the as-prepared photocatalysts is XM/P25, where X refers to the nominal metal loading (1 or 5 wt %) and M denotes Cu or Ni. For thermally reduced photocatalysts, the letter ″r″ is appended (i.e., XM/P25-r).

Scheme 1 summarizes the synthesis route and the photocatalysts obtained, which have been thoroughly characterized, as detailed in the next section.

**Scheme 1:**
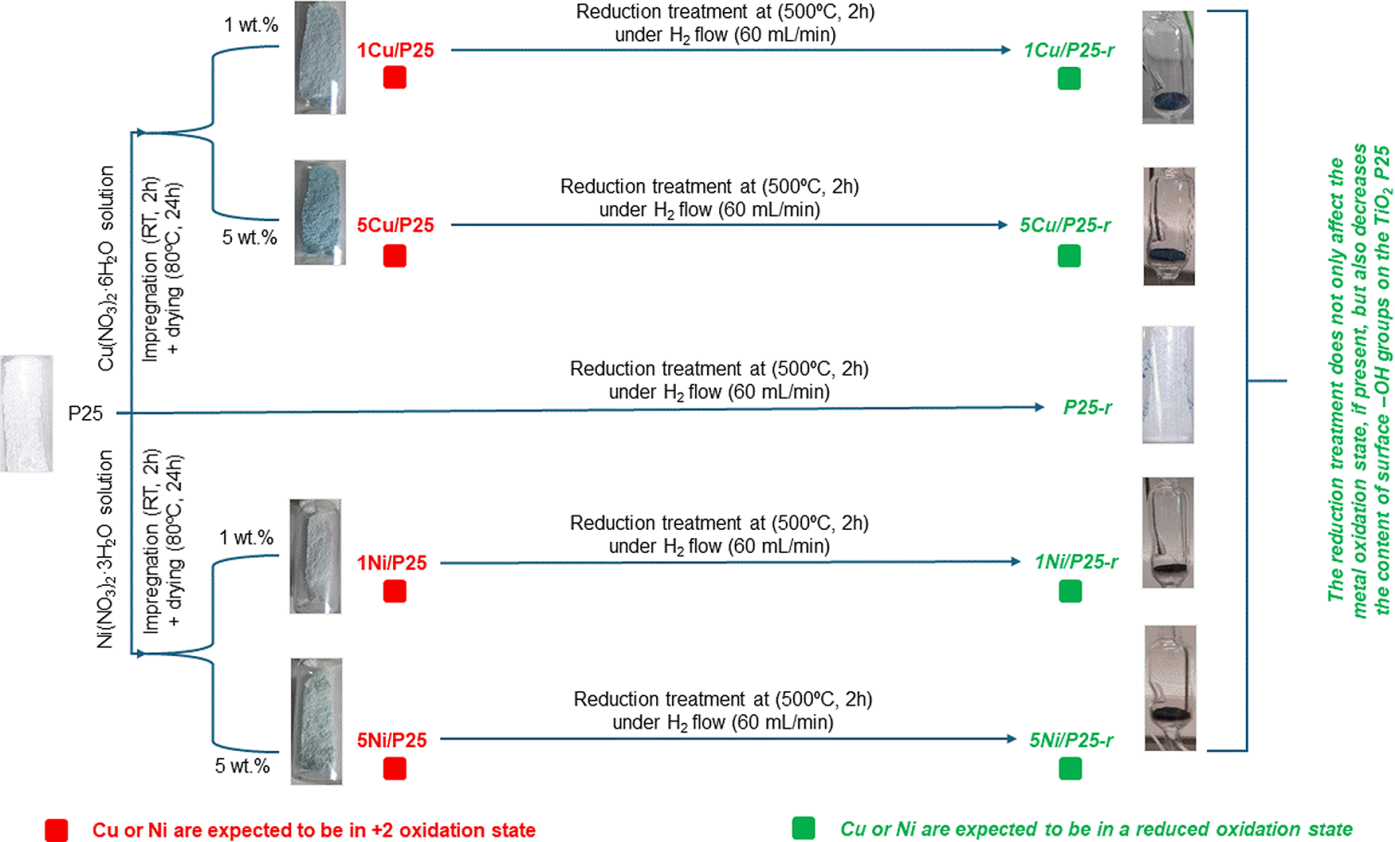
Scheme of the Synthesis Conditions and Chemical Composition of the Studied Photocatalysts

### 2.2 Characterization of the Photocatalysts

The impact of the metal incorporation on the crystallinity of TiO_2_, as well as the presence and crystallinity of metallic phases in the XM/P25 and XM/P25-r photocatalysts, was analyzed using X-ray diffraction (XRD). For comparison, P25 and P25-r were also examined. XRD patterns were acquired using a Rigaku Miniflex II, operating at 30 kV and 15 mA with Cu Kα radiation. Scans were performed at a rate of 2°/min across a 2θ range of 6 to 80°.

The actual metal content in the XM/P25 photocatalysts was determined by inductively coupled plasma optical emission spectrometry (ICP-OES). A small amount of each photocatalyst was dissolved in concentrated aqua regia (HCl and HNO_3_ in a 3:1 ratio) and subjected to ultrasonic treatment at 80 °C for 45 min to ensure complete dissolution. After filtration and dilution, the solutions were analyzed using a Perkin Elmer 7300 DV equipment, targeting the emission lines at 324.750 nm for Cu and 231.604 nm for Ni.

The textural properties of all the photocatalysts were evaluated by N_2_ adsorption–desorption at −196 °C using a Nova 800 instrument. Before the analysis, the samples were degassed at 250 °C for 4 h. The specific surface area (*S*_BET_) was calculated using the BET equation for *p*/*p*_0_ values between 0.05 and 0.30. The micropore volume (V_DR N_2__) was determined using the Dubinin–Radushkevich equation for *p*/*p*_0_ values between 0.01 and 0.10. The mesopore volume (*V*_meso_) was estimated as the difference between the adsorbed N_2_ volumes at *p*/*p*_0_ values of 0.90 and 0.20. The total pore volume (*V*_Total_) was obtained from the adsorbed N_2_ volume at *p*/*p*_0_ = 0.99.^20^

The distribution of metal species in XM/P25 and XM/P25-r was assessed using Transmission Electron Microscopy (TEM) with a FEI Talos F200X G2 microscope operating at 200 kV. The average particle size of the metal species was calculated using the J-Image software, selecting a minimum number of 30 particles to analyze for 1Cu/P25, 5Cu/P25, 5Cu/P25-r, and 5Ni/P25-r. Only 14 particles were analyzed for 1Cu/P25-r. For the rest of photocatalysts, 1Ni/P25, 5Ni/P25, and 1NiP25-r, the particle size measurement was not possible due to their small size and/or difficult identification, and the mean size could not be estimated.

Elemental composition analysis was carried out using a FEI Talos F200X G2 microscope equipped with energy dispersive spectroscopy (EDS), also operating at 200 kV. This combination of TEM and EDS provided detailed insights into both the morphology and spatial distribution of the metal species on the TiO_2_ surface.

The surface composition of all the studied photocatalysts was examined by X-ray photoelectron spectroscopy (XPS), using a Thermo-Scientific NEXSA G2 instrument with Al Kα X-ray radiation (1486.6 eV) under ultrahigh-vacuum conditions. Key parameters included a pass energy of 50 eV, a scan step of 0.1 eV, and sample irradiation within an ellipsoidal area with a major axis of 400 μm. The binding energies (B.E.) were calibrated using the C 1s transition at 284.6 eV. Additionally, Auger spectroscopy analysis was performed to differentiate Cu^0^ and Cu^+^.

The surface composition of P25 and its thermally reduced counterpart P25-r, specifically focusing on hydroxyl (−OH) groups, was analyzed through thermogravimetric (TG) analysis, aiming to confirm if the surface chemistry was modified by the thermal treatment in hydrogen. Measurements were conducted in a SDT Q600 thermobalance (from TA Instruments), using about 10 mg sample. The analysis conditions included a N_2_ flow rate of 100 mL/min following a three-stage temperature program: initial heating at 10 °C/min to 120 °C with 15 min isothermal period, followed by continuous heating at 10 °C/min up to 750 °C. Metal-containing TiO_2_ photocatalysts, as prepared or after thermal reduction treatment, were not analyzed due to potential interference from metal species.

Optical properties were investigated using UV–vis Diffuse Reflectance Spectroscopy (UV–vis DRS) with a Jasco V-670 spectrophotometer, in the200–800 nm wavelength range. Approximately 0.1 g of the sample was analyzed, with BaSO_4_ used as the reference material. The band gap energy (*E*_g_) was calculated using a Tauc plot, applying the Kubelka–Munk function (*F*(*R*) × *h*)^1/2^ versus photon energy (*h*ν), following the indirect transition method^21^.

The efficiency of metal incorporation in reducing the electron–hole pairs recombination in the photocatalysts after UV excitation was evaluated via photoluminescence (PL) spectroscopy. Spectra were recorded with a PicoQuant Fluotime 250 fluorescence lifetime spectrometer. Measurements were performed at room temperature using a 373.5 nm excitation wavelength, provided by a diode laser (LDH–P-C-373) and a 400 nm filter. Emission spectra were collected over the 385–885 nm range.

### 2.3 Photocatalyzed Water Splitting Tests and Analysis of the Produced H_2_

The experimental setup used to perform the UV-photocatalyzed WS tests consists of a 1 L cylindrical glass reactor equipped with a TQ-150 undoped UV lamp (150 W consumed, 47 W irradiated, λ_max_ = 365 nm; see the irradiation profile in Figure S1a in the Supporting Information) located inside the reactor. The jacket of the lamp is quartz-based. The system was coupled to a mass spectrometer (Omnistar GDS201 01, Pfeiffer vacuum) for real-time gas analysis. For each test, 20 mg of photocatalyst (P25, P25-r, XM/P25, or XM/P25-r) was suspended in 500 mL of distilled water. Before the experiments, the reaction medium was stirred and purged with He (30 mL/min) in the dark for 2 h to remove any dissolved oxygen. Maintaining the He flow, the lamp was then switched on, and the irradiation was maintained for 5 h. The generated hydrogen gas was continuously swept from the reactor by the He flow and directed to the mass spectrometer for analysis. Quantification of the produced H_2_ was performed using a calibrated gas cylinder containing 500 ppm of H_2_. The H_2_ production results were reported as total micromoles (μmol) generated over 5 h, or as μmol per gram of catalyst per hour, μmol·g_cat_^–1^·h^–1^. Oxygen (O_2_) gas evolution was also assessed but not quantified. A blank experiment, without any photocatalyst, was also performed for comparison.

An extended experiment with a total UV irradiation time of 15 h was conducted using the 5Cu/P25 photocatalyst. This experiment consisted of three consecutive 5 h UV irradiation cycles. Between cycles, the UV lamp was turned off, and the photocatalyst suspension in water was kept under stirring in the dark under an inert atmosphere for, approximately, 19 h before starting the next 5 h irradiation cycle.

In addition, visible light testing was performed using the 5Cu/P25-r photocatalyst, exploring its potential for solar-driven hydrogen production. The visible light was provided by a TXE150 Xe lamp (150 W power consumption, with a peak wavelength around λ _max_ = 875 nm; see the irradiation profile in Figure S1b in the Supporting Information).

## 3 Results and Discussion

### 3.1 Characterization of the Photocatalysts

Figure S2 in the Supporting Information shows photographs of all the photocatalysts. The XRD patterns for all the studied photocatalysts are presented in Figure 1. In Figure 1a, diffraction peaks corresponding to anatase (A) and rutile (R) are clearly identified.^22^ However, no distinct peaks associated with Cu or Ni species are detected, even at the higher metal loading of 5 wt %. This absence of metal-related peaks can be attributed to: (1) the low metal concentration, (2) the small size and/or high dispersion of metal species, and/or (3) the amorphous nature of these particles. Previous studies have suggested that metallic species can partially integrate into the TiO_2_ lattice, particularly when co-synthesis is used for the preparation of metal-TiO_2_ composites, with the metal and Ti precursors.^22^ However, in this study the metals were incorporated via mild-temperature impregnation onto pre-synthesized and stable TiO_2_ (P25), rather than being co-synthesized, making lattice incorporation of the metal species unlikely.

**Figure 1:**
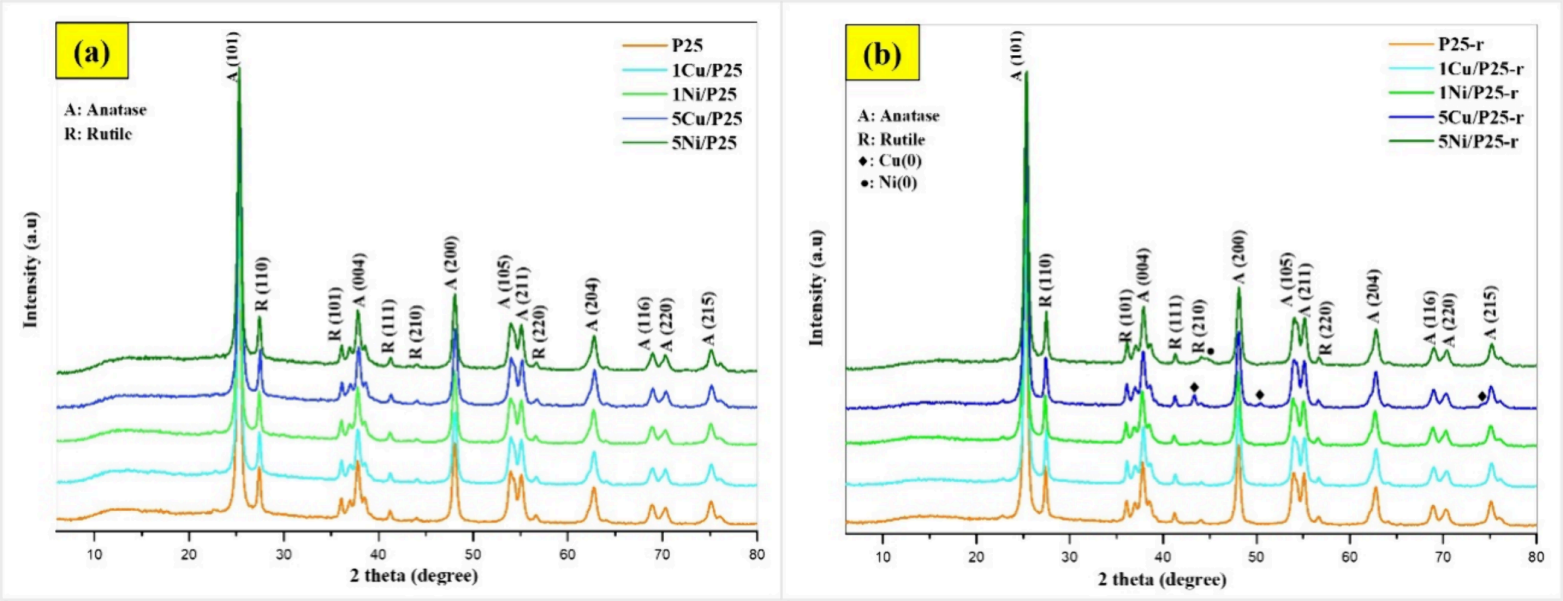
XRD patterns of the photocatalysts: (a) P25 and XM/P25, and (b) P25-r and XM/P25-r.

In Figure 1b, in addition to anatase and rutile peaks, small and broad peaks appear for the XM/P25-r photocatalysts, corresponding to metallic phases formed as a result of the reduction process. The fact that no metallic domains are detected in the XRD patterns of the XM/P25 photocatalysts, whereas they are in the patterns of the XM/P25-r ones, would imply that the heat treatment involved in the preparation of the XM/P25-r samples results in metallic domains which are either larger in size and/or more crystalline.

For 5Cu/P25-r, three distinct peaks are observed, at approximately 43.3, 50.4, and 74.3°, which correspond to the (111), (200), and (220) planes of the face-centered cubic (fcc) structure of metallic copper.^23^ In the case of 5Ni/P25-r, a single small and broad peak appears at 44–45°, likely corresponding to the (111) plane of the fcc structure of metallic Ni.^24^ These results remark that the amounts of Cu and, especially, Ni in 1M/P25-r are small and/or the metals are present in either a low crystallinity state and/or are highly dispersed.

No significant differences are observed in the intensity and width of the TiO_2_ peaks between P25 and the other photocatalysts (XM/P25, P25-r, and XM/P25-r). This indicates that the incorporation of metal precursors and/or the reduction treatment do not significantly alter the phase composition^25^ or the crystallite size of the TiO_2_ phases.^26^ Additionally, no shifts in the peak positions of the anatase and rutile phases are evident, suggesting that the deposited Cu or Ni-species, even after thermal reduction, are not incorporated into the lattice of TiO_2_ but are likely located on the surface of the TiO_2_ particles.^27^

The actual metal loading in the XM/P25 photocatalysts (XCu/P25 and XNi/P25), as determined by ICP-OES, was found to be 1.11 wt % for Cu or 0.91 wt % for Ni when X = 1, and 4.81 wt % for Cu or 4.35 wt % for Ni when X = 5. These values reveal a close match between the nominal and actual metal contents, strongly supporting the success of the impregnation procedure.

The N_2_ adsorption–desorption isotherms at −196 °C of all the studied photocatalysts, presented in Figure S3 in the Supporting Information ((a) XM/P25 and (b) XM/P25-r photocatalysts), are classified as type IV according to the IUPAC, with a hysteresis loop typical of mesoporous materials.^28^ The incorporation of metallic species, as well as the reduction treatment, only results in a slight reduction in the surface area, along with a minor decrease in the total pore volume compared to P25. No significant changes are noted in the volume of micropores (V_DR‑N2_) or in the volume of mesopores (*V*_meso_) (see Table S1 in the Supporting Information). This suggests that the metal incorporation and/or the reduction treatment have a minimal impact on the overall porous structure of the photocatalysts.

TEM bright-field images of the metal-containing photocatalysts are shown in Figure 2. For the XM/P25 photocatalysts, Figure 2a shows that in 1Cu/P25 sample, Cu particles are well-dispersed and homogeneously distributed on the TiO_2_ surface. A similar distribution is observed in the 5Cu/P25 catalyst (Figure 2b). The average size of the Cu particles is below 3 nm, as shown in the particle size distribution graphs in Figure S4 of the Supporting Information.

**Figure 2:**
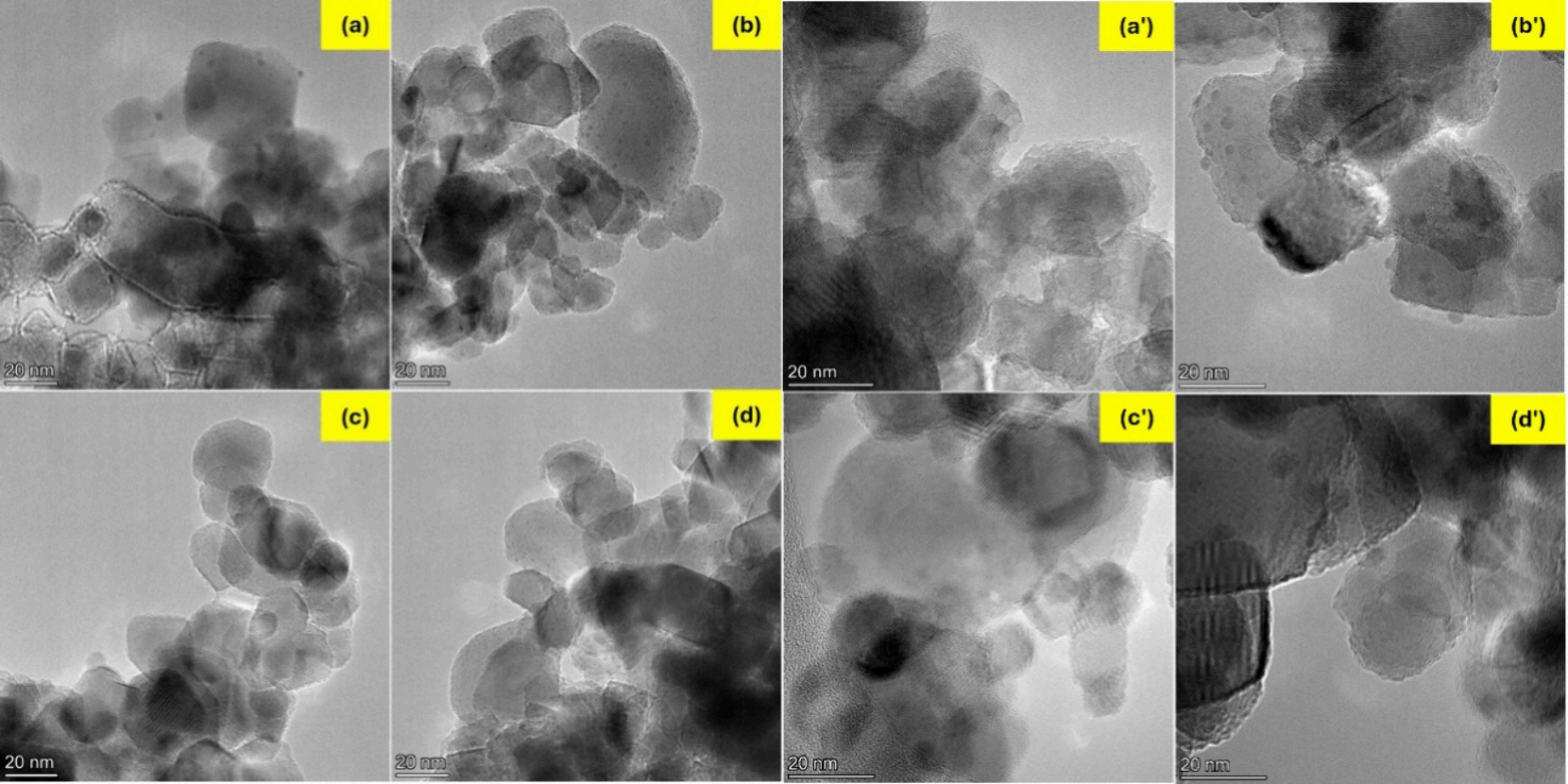
TEM images of the XM/P25 photocatalysts: (a) 1Cu/P25, (b) 5Cu/P25, (c) 1Ni/P25, and (d) 5Ni/P25; and XM/P25-r: (a′) 1Cu/P25-r, (b′) 5Cu/P25-r, (c′) 1Ni/P25-r, and (d′) 5Ni/P25-r. Images were obtained using a FEI Talos F200X G2 microscope operating at 200 kV.

In contrast, in 1Ni/P25 and 5Ni/P25 (Figure 2c, d), Ni particles are difficult to observe, despite the similar atomic number for Ni and Cu, and similar expected contrast between the metals and TiO_2_, suggesting that the Ni particles are, in general, smaller than the Cu ones.

TEM images also confirm a good dispersion and homogeneity of Cu particles on the TiO_2_ surface for both 1Cu/P25-r and 5Cu/P25-r (Figure 2a′, b′), with mean particle sizes of 41 and 31 nm in 1Cu/P25-r and 5Cu/P25-r, respectively (Figure S4). For the Ni-containing samples (Figures 2c′, d′), both small and large particles are observed, particularly in 5Ni/P25-r. While particle size measurement remains challenging for 1Ni/P25-r, the mean particle size for 5Ni/P25-r is approximately 48 nm (Figure S4e). These results indicate a significant increase in metal particle size after the hydrogen reduction thermal treatment at 500 °C, suggesting that the heat treatment promotes the metallic particles mobility, leading to sintering.^29^

Figure 3 presents EDS mapping analysis, providing further insights about the distribution of Cu or Ni species on the TiO_2_ surface. In XCu/P25, a uniform distribution of Cu is shown, with minimal agglomeration in 1Cu/P25, while 5Cu/P25 exhibits some clustering. 1Ni/P25 shows a uniform distribution of Ni species, resembling the Cu distribution in 1Cu/P25. In contrast, localized clustering is observed in 5Ni/P25.

**Figure 3:**
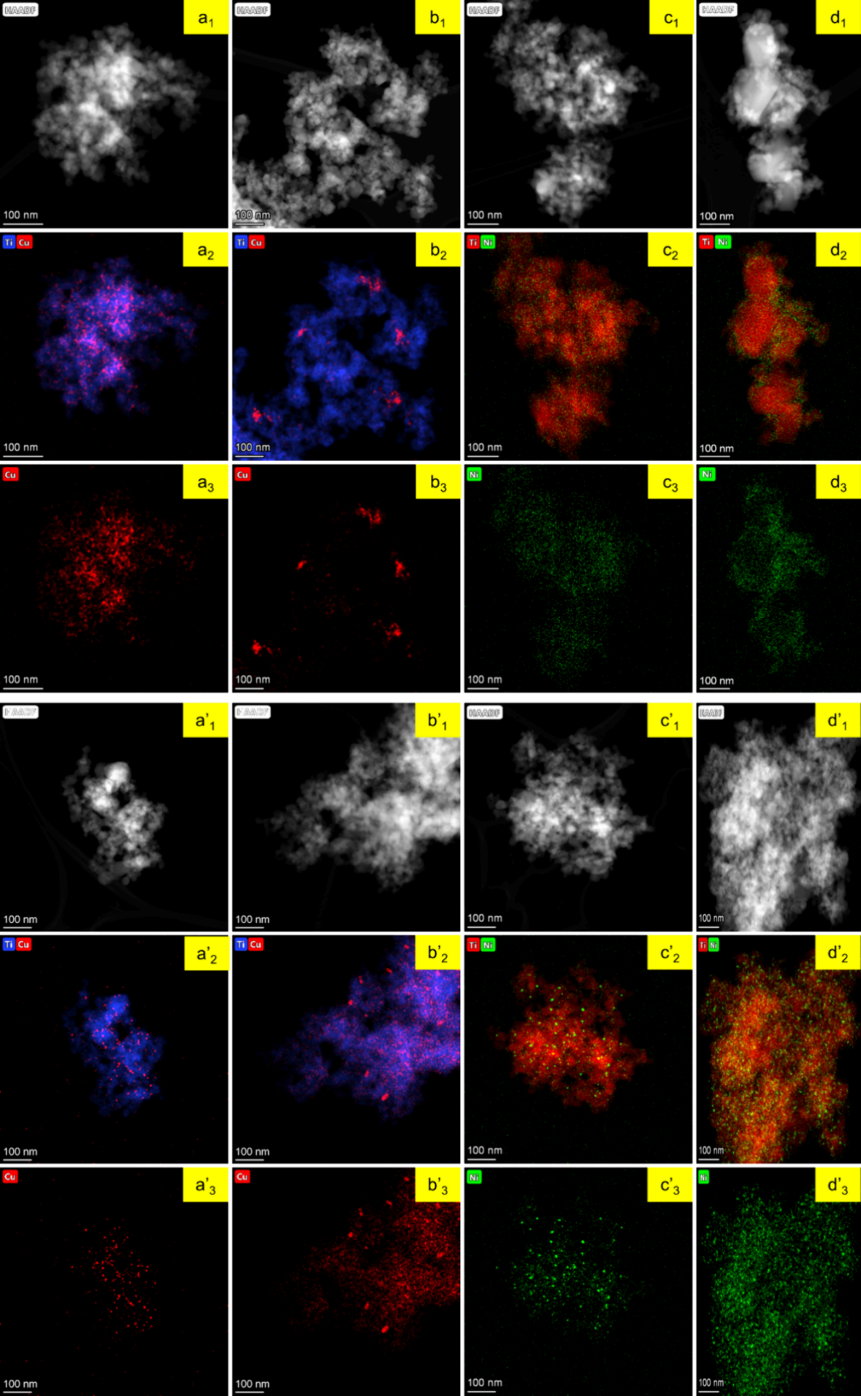
Dark field TEM images and EDS mapping for XM/P25 photocatalysts: (a1, a2, a3: 1Cu/P25), (b1, b2, b3: 5Cu/P25), (c1, c2, c3: 1Ni/P25), and (d1, d2, d3: 5Ni/) and XM/P25-r photocatalysts (a1′, a2′, a3′: 1Cu/P25-r), (b1′, b2′, b3′: 5Cu/P25-r), (c1′, c2′, c3′: 1Ni/P25-r), and (d1′, d2′, d3′: 5Ni/P25-r).

For the XM/P25-r photocatalysts, EDS maps confirm a uniform distribution of Cu or Ni species in 1M/P25-r, with minimal agglomeration, as in the as-prepared samples. However, in 5M/P25-r, increased agglomeration is evident, although overall coverage remains satisfactory. This analysis corroborates the observed significant increase in particle size for XM/P25-r compared to XM/P25, which has been attributed to potential sintering during the reduction process.

XPS analysis data of Ti and O (presented in Figures S5 and S6 and Tables S2 and S3), as well as Cu or Ni (presented in Figure 4 and Table 1), reveal key surface transformations after metal incorporation and thermal reduction treatment.

**Figure 4:**
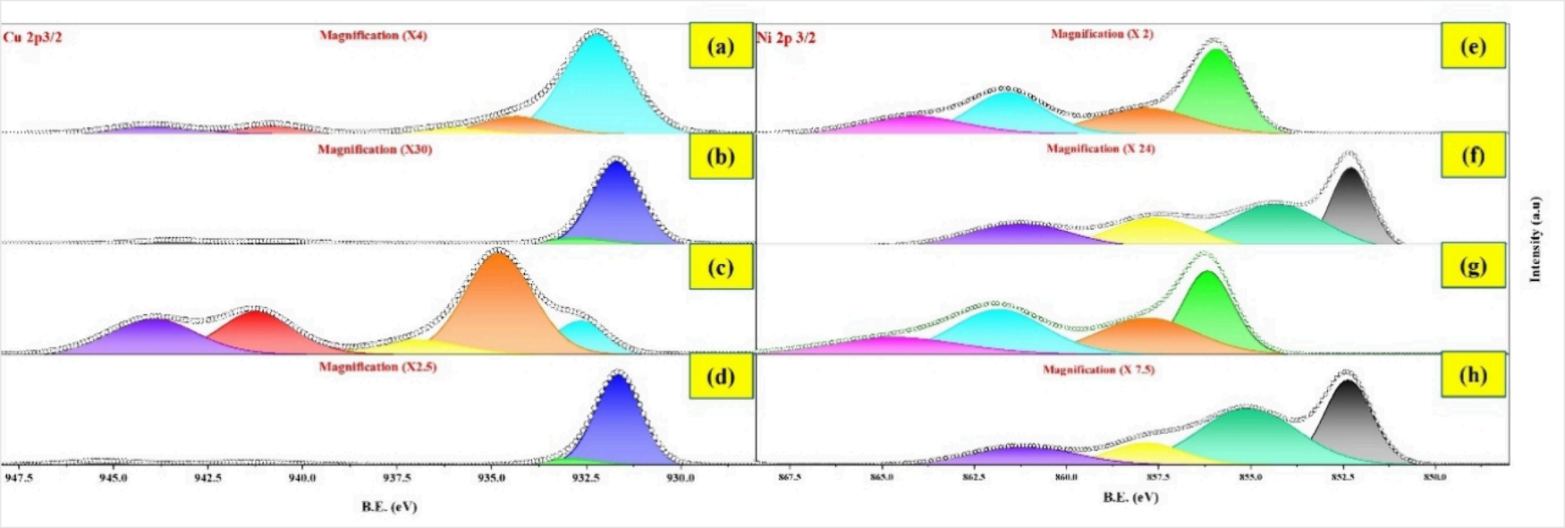
Cu or Ni 2p3/2 XPS spectra for XM/P25 photocatalysts ((a) 1Cu/P25, (c) 5Cu/P25, (e) 1Ni/P25, and (g) 5Ni/P25) and XM/P25-r photocatalysts ((b) 1Cu/P25-r, (d) 5Cu/P25-r, (f) 1Ni/P25-r, and (h) 5Ni/P25-r).

**Table 1:** XPS Data of Cu or Ni in XM/P25 and XM/P25-r: Binding Energy (Cu 2p3/2 or Ni 2p3/2), Oxidation State/s, Proportion of Metal Species, and Surface Metal Content (M)

Photocatalyst	Binding energy (eV)	Oxidation state	Proportion (%)	M (wt %)
1Cu/P25	932.2	Cu^+^	68	4.9
	934.4	Cu^2+^	32	
	936.3			
5Cu/P25	932.6	Cu^+^	11	20.2
	934.8	Cu^2+^	89	
	937.0			
1Cu/P25-r	931.7	Cu^0^	82	1.0
	932.8	Cu^2+^	18	
5Cu/P25-r	931.6	Cu^0^	80	2.2
	933.0	Cu^2+^	20	
1Ni/P25	855.9	Ni^2+^	100	4.7
	857.9			
5Ni/P25	856.2	Ni^2+^	100	11.7
	857.8			
1Ni/P25-r	852.3	Ni^0^	26	0.8
	854.3	Ni^2+^	74	
	857.6			
5Ni/P25-r	852.4	Ni^0^	30	3.1
	855.1	Ni^2+^	70	
	857.8			

The XPS analysis of Ti 2p3/2 shows significant interactions with Cu or Ni after metal incorporation and/or thermal reduction. In P25, the Ti 2p3/2 peak corresponding to Ti^4+^ is observed at 458.55 eV.^30^ Incorporating Cu or Ni (1 or 5 wt %) to P25 induces slight upward shifts in Ti 2p3/2 peaks in XM/P25, indicating interactions between the TiO_2_ and the metal species.^31^ Specifically, Ti 2p3/2 peaks shift to 458.71 eV for 1Cu/P25, 458.96 eV for 5Cu/P25, 458.75 eV for 1Ni/P25, and 458.85 eV for 5Ni/P25.

In P25-r, the peaks corresponding to Ti^4+^ slightly shift to 459.04 eV, reflecting changes associated with thermal treatment, as reported in previously published data.^25^ In XM/P25-r, further shifts exist in Ti 2p3/2 peaks. For Cu-modified samples, Ti 2p3/2 binding energy decreases, with peaks at 458.36 eV for 1Cu/P25-r and 458.59 eV for 5Cu/P25-r. Notably, a peak at 457.58 eV in 5Cu/P25-r is indicative of the existence of Ti^3+^.^32^ For XNi/P25-r photocatalysts, the Ti 2p3/2 binding energy shifts are minimal compared to their XNi/P25 counterparts, with peaks observed at 458.98 eV for 1Ni/P25-r and 458.99 eV for 5Ni/P25-r. The decrease in binding energy and the formation of Ti^3+^ (in the case of copper) provide strong evidence for enhanced metal-TiO_2_ interactions, consistent with the strong metal–support interaction (SMSI) effect.^33^

The O 1s spectra reveal variations in the distribution of oxygen species. In P25, the O 1s spectrum shows contributions of lattice oxygen at 529.78 eV (90%) and surface hydroxyl groups at 531.19 eV (10%).^32^ For the XM/P25 photocatalysts, lattice oxygen is observed at 530.00 eV with 80% content for 1Cu/P25, 530.19 eV with 71% content for 5Cu/P25, 529.99 eV with 81% content for 1Ni/P25, and 530.07 eV with 68% content for 5Ni/P25. The peaks corresponding to hydroxyl groups^34^ , around 531 eV,^35,36^ remain consistent across XM/P25 samples, with contents ranging from 6 to 7%. Peaks in this region may also include contributions from Cu–O/Ni–O species. Higher-energy peaks (532.74–533.05 eV), attributed to adsorbed water,^35^ are prominent in XM/P25 (12–24%) but absent in P25, indicating incomplete dehydration during the drying process applied to the metal-containing materials during their preparation and/or the presence of adsorbed moisture.

In the reduced samples, the O 1s spectrum of P25-r closely resembles that of P25, with a 530.26 eV peak for lattice oxygen (90%) and surface species at 531.39 eV (9%). The minor peak observed at 532.66 eV (1%) can be attributed to adsorbed moisture due to air exposure after the thermal reduction process. In XM/P25-r samples, the O 1s spectra exhibit notable changes. For Cu-modified samples, a decrease in binding energy is observed for 1Cu/P25-r (529.53 eV with 95% content), while a lower-energy peak at 528.46 eV appears in 5Cu/P25-r, indicating the formation of oxygen vacancies and strong Cu–TiO_2_ interactions. In Ni-modified samples, the O 1s peaks shift to higher energies for increasing Ni content, suggesting distinct Ni–TiO_2_ interactions.

The content of hydroxyl groups, particularly in XCu/P25-r, is 5%, which is slightly lower than in XCu/P25 samples. Furthermore, minor shifts in peak positions are observed for the XCu/P25-r samples: the peak for 1Cu/P25 shifts from 531.18 to 530.97 eV after reduction, while for 5Cu/P25 it shifts from 531.24 to 531.68 eV. These shifts further support the formation of oxygen vacancies upon reduction. For Ni-containing samples, the peak position of 1Ni/P25-r (531.30 eV) is similar to that of 1Ni/P25 (531.26 eV), while a slight change is observed for 5Ni/P25-r (531.33 eV compared to 531.53 eV in 5Ni/P25).

The O/Ti ratios (2.83 for 1Cu/P25, 3.15 for 5Cu/P25, 2.49 for 1Ni/P25, and 3.34 for 5Ni/P25) are related to the presence of adsorbed water, especially at higher (5 wt %) metal loadings. After thermal reduction, a decrease in the O/Ti ratios indicates a reduction in surface oxygen species content. While the O/Ti ratio drops from 2.63 in P25 to 2.57 in P25-r, the decrease is more pronounced in metal-modified samples after reduction, particularly at higher metal loadings. For example, the O/Ti ratio decreases to 2.29 for 1Cu/P25-r and 2.16 for 5Cu/P25-r, reflecting reduced surface hydroxylation and lower levels of adsorbed water.

Residual nitrates from Cu­(NO_3_)_2_ and Ni­(NO_3_)_2_ precursors are indicated by N 1s peaks at 406.97–407.25 eV (data not shown).^30^ These peaks are associated with the incomplete decomposition of metallic nitrate precursors during the low-temperature drying step (80 °C) without additional heat treatment. Following the reduction treatment in hydrogen, N 1s peaks disappear, confirming complete nitrate removal during the high-temperature thermal reduction (500 °C).

For the XCu/P25 samples, the Cu 2p3/2 spectra (Figure 4a, c) reveal a mixture of Cu^2+^ species with Cu^0^/ Cu^+^ (or both)^37,38^ along with shake-up satellite peaks.^37,38^ In 1Cu/P25, copper primarily exists as Cu^0^/Cu^+^ or both, whereas 5Cu/P25 predominantly contains Cu^2+^ (89%). After thermal reduction (Figure 4b, d), XPS peaks shift to lower energy values, indicating the presence of reduced species. Peaks near ∼932 eV can be attributed to Cu^0^ or a mixture of Cu^0^ and Cu^+^,^37^ while a small peak near 933 eV suggests the minor presence of Cu^2+^. These are accompanied by very small satellite peaks in the 940.7–945.3 eV range.^33^ Both Cu^0^ and Cu^+^ peaks typically appear in a similar binding energy range, making their distinction challenging.

For XNi/P25 samples, the Ni 2p3/2 spectra (Figure 4e, g) reveal multiple Ni^2+^ environments, with peaks in the 855–864 eV range and associated satellite features, indicating varied surface interactions.^39^ Upon reduction (Figure 4f, h), lower-energy peaks appear near 852 eV, corresponding to Ni^0^,^39^ while peaks in the 854–857 eV range indicate the presence of Ni^2+^ species.^39^ These are accompanied by a satellite peak at 861.3 eV. The proportion of Ni^0^ increases to 26% in 1Ni/P25-r and 30% in 5Ni/P25-r.

To differentiate between Cu^0^ and Cu^+^ oxidation states in the Cu-containing photocatalysts, Auger spectra were also analyzed. This analysis is challenging due to the intense XPS Ti 2s transition at 565.3 eV, which overlaps with the Auger Cu LMM transition (which occurs between 567.6 and 573.6 eV). The main features in the Cu (L_3_M_4,5_M_4,5_) Auger spectra appear in the 912–922 eV range, with Cu^0^ contributing at 918.61 eV, Cu_2_O at 916.99 eV, CuO at 917.57 eV, Cu (OH)_2_ at 916.3 eV, and Cu (NO_3_)_2_ at 914.98 eV.^37^

A thorough analysis of the Auger spectra (Figure S7 in the Supporting Information) reveals significant differences between samples, particularly among 5Cu/P25 and 5Cu/P25-r and P25. In the as-prepared samples, the signal intensity in the range of 912–918 eV for 1Cu/P25 is slightly higher than that of P25 (see Figure S7a), while for 5Cu/P25 a more pronounced increase is shown (see Figure S7b), confirming the presence of various copper compounds. These include Cu­(NO_3_)_2_ and, more notably, Cu­(OH)_2_ and Cu_2_O, with the peak intensity being more pronounced in their respective regions. For both samples, a shift of the main peak to lower values, by 0.35 and 0.90 eV for 1Cu/P25 and 5Cu/P25, respectively, compared to the position of P25 (921.9 eV), indicating strong Cu–Ti interactions.

After thermal reduction treatment, the emission spectrum of 1Cu/P25-r (Figure S7c) shows lower intensity compared to P25, making the interpretation more challenging. However, upon examining the 5Cu/P25-r spectrum (Figure S7d), it is evident that the intensity is significantly higher than that for P25 from 918 eV, indicating the presence of Cu in its reduced form, particularly Cu^0^. Comparing 5Cu/P25 and 5Cu/P25-r, there are noticeable differences in the spectra, confirming that the thermal treatment has a significant effect on the Cu species, altering their chemical state. The shift of the main signal to lower values is minimal, approximately 0.14 eV for 1Cu/P25-r and negligible for 5Cu/P25-r, compared to P25.

Thus, Auger analysis allows to effectively differentiate between Cu^0^ and Cu^1+^. In the as-prepared XCu/P25 photocatalysts, Cu^1+^ is confirmed, while Cu^0^ is not prominently observed. This aligns with the preparation conditions used in this study (impregnation followed by drying at 80 °C), being thermodynamically unlikely the formation of metallic Cu^0^. Conversely, in the thermally reduced samples, Cu^0^ is evident, whereas Cu^1+^ is not detected. Notably, in the 1Cu/P25-r sample, the signal for Cu^0^ is less distinct due to the low copper content (see Table 1), also consistent with the XRD results.

The combined information from XPS and Auger spectroscopy indicates that the oxidation states of copper in the XCu/P25 photocatalysts are +1 and +2, while Cu is predominantly in the 0-oxidation state in the XCu/P25-r samples.

Table 1 compiles the XPS and Auger data for Cu and Ni in XM/P25 and XM/P25-r samples, including binding energies (Cu 2p3/2 or Ni 2p3/2), oxidation states, proportion of metal species, and surface metal content. In XM/P25 samples, copper primarily exists as Cu^2+^ and Cu^+^, indicating some change in its chemical state compared to the original copper­(II) nitrate precursor used for the impregnation. Notably, the proportion of Cu^+^ is higher in 1Cu/P25 than in 5Cu/P25. This can be explained by the higher metal dispersion at lower metal loadings, which facilitates better contact and interaction between the metal and the TiO_2_ surface. Specifically, the proportion of reduced or partially reduced copper species is 68% for 1Cu/P25 and only 11% for 5Cu/P25. Ni in XM/P25 samples predominantly exists in the Ni^2+^ state.

As anticipated, thermal reduction results in materials with significant content of reduced metallic phases, specifically in XCu/P25-r (82% of copper in the reduced state for 1Cu/P25-r and 80% for 5Cu/P25-r), while XNi/P25-r demonstrated significantly lower content (e.g., 26% for 1Ni/P25-r). The binding energy (B.E.) values for the thermally reduced photocatalysts are slightly shifted to lower values compared to those reported in the literature.^30,37^ For instance, shifts of 1.0 and 0.9 eV in the Cu^0^ peak are observed for 1Cu/P25-r and 5Cu/P25-r, respectively, and 0.4 and 0.3 eV in the Ni^0^ peak for 1Ni/P25-r and 5Ni/P25-r, respectively. These shifts further support the presence of SMSI, aligning with similar systems reported in the literature.^33,39^ Notably, the B.E. shifts are more pronounced in the XCu/P25-r photocatalysts compared to the XNi/P25-r ones. Regarding the surface metal content, the XM/P25 photocatalysts show significant metal surface enrichment, particularly when X = 5, with 20.2% for 5Cu/P25 and 11.7% for 5Ni/P25. This is consistent with the higher surface coverage observed in TEM-EDS maps. After thermal reduction, a significant decrease in surface metal content is evident. This reduction is likely due to particle sintering,^29^ as indicated by the larger particle sizes and agglomeration observed in TEM images and TEM-EDS maps. Additionally, this decrease may result from the ″decoration″ effect of TiO_2_ over the metal particles, associated with SMSI. Such SMSI effects can partially encapsulate the metal particles, reducing their accessibility, as reported in previous studies.^40^

Overall, XPS and Auger analyses indicate that modifying TiO_2_ with Cu or Ni introduces specific surface interactions and transformations. These interactions lead to: (1) changes in the electronic structure of Ti atoms, evidenced by shifts in the binding energy of Ti 2p3/2 peaks; (2) alterations in the distribution of oxygen species on the surface; and (3) variations in surface hydroxylation and hydration. The extent of these effects depends on both the type of metal and its loading, with higher metal loadings generally resulting in more pronounced changes. Specifically, XCu/TiO_2_ photocatalysts exhibit stronger surface interactions than their Ni-modified counterparts. As a result, Cu undergoes reduction from Cu^2+^ to Cu^+^, while Ni predominantly remains in the Ni^2+^ state. After thermal reduction, Cu modification also induces stronger TiO_2_ interactions than Ni, promoting the formation of oxygen vacancies and the presence of Ti^3+^.

Data from the TG analysis of P25 and P25-r are presented in Figure S8 and Table S4, both in the Supporting Information. Following the methodology reported by Mueller et al., mass loss was measured and analyzed across several temperature intervals to identify the removal of various surface species.^41^ Specifically, the mass loss observed between 30 and 120 °C corresponds to the removal of physisorbed water (humidity), whereas the interval from 120 to 300 °C is associated with the removal of weakly bonded hydroxyl (–OH) groups. The mass loss between 300 and 600 °C corresponds to the removal of strongly bonded–OH groups.

The results indicate a slight decrease in mass loss for P25-r compared to P25 in the 30 to 120 °C range (0.62% vs 0.65%), consistent with the removal of physisorbed water during the reduction process, which involves heating under hydrogen flow. In the 120 to 300 °C range, P25-r also shows a lower mass loss compared to P25 (0.45% vs 0.50%), suggesting a reduced content of weakly bonded −OH groups in P25-r. Furthermore, in the 300 to 600 °C range, P25-r exhibits a notably lower mass loss than P25 (0.12% vs 0.28%), indicating a significant reduction in strongly bonded–OH groups in P25-r concerning P25. The substantial difference in total −OH content between these two (0.78% for P25 vs 0.57% for P25-r) supports that thermal reduction in a hydrogen atmosphere alters the hydroxyl surface chemistry of TiO_2_. This observation aligns with XPS analysis, which shows a slightly lower O/Ti ratio for P25-r compared to P25, suggesting a decrease in the content of surface oxygen-containing species, including −OH groups, after applying a thermal treatment under H_2_ gas flow.

The TG analysis was performed only on the non-metal-containing TiO_2_ photocatalysts (P25 and P25-r), as the presence of metal species could complicate the interpretation. However, the reduction in the content of oxygen surface species, including −OH groups, through thermal treatment under H_2_ gas flow, is similarly proposed for these photocatalysts.

UV–vis DRS absorption spectra of the studied photocatalysts are displayed in Figure 5. Figure 5a shows that the absorbance of the XM/P25 photocatalysts is consistently higher than that of P25 across the entire wavelength range (λ), with more pronounced differences at wavelengths greater than 500 nm. In this region, significant variations are noted: XCu/P25 shows higher absorbance than XNi/P25, and 5M/P25 exhibits greater absorbance than 1M/P25. Specifically, 1Cu/P25, with its lighter blue color (see Figure S2), demonstrates higher absorbance than 1Ni/P25, which displays a light green color. Similarly, 5Cu/P25, with its more intense blue color, shows much higher absorbance than 5Ni/P25, which has an intense green color (see Figure S2). This indicates that Cu-containing photocatalysts, especially at higher metal loadings, exhibit superior light absorption than their Ni-based counterparts, likely due to more efficient electronic transitions in Cu species.

**Figure 5:**
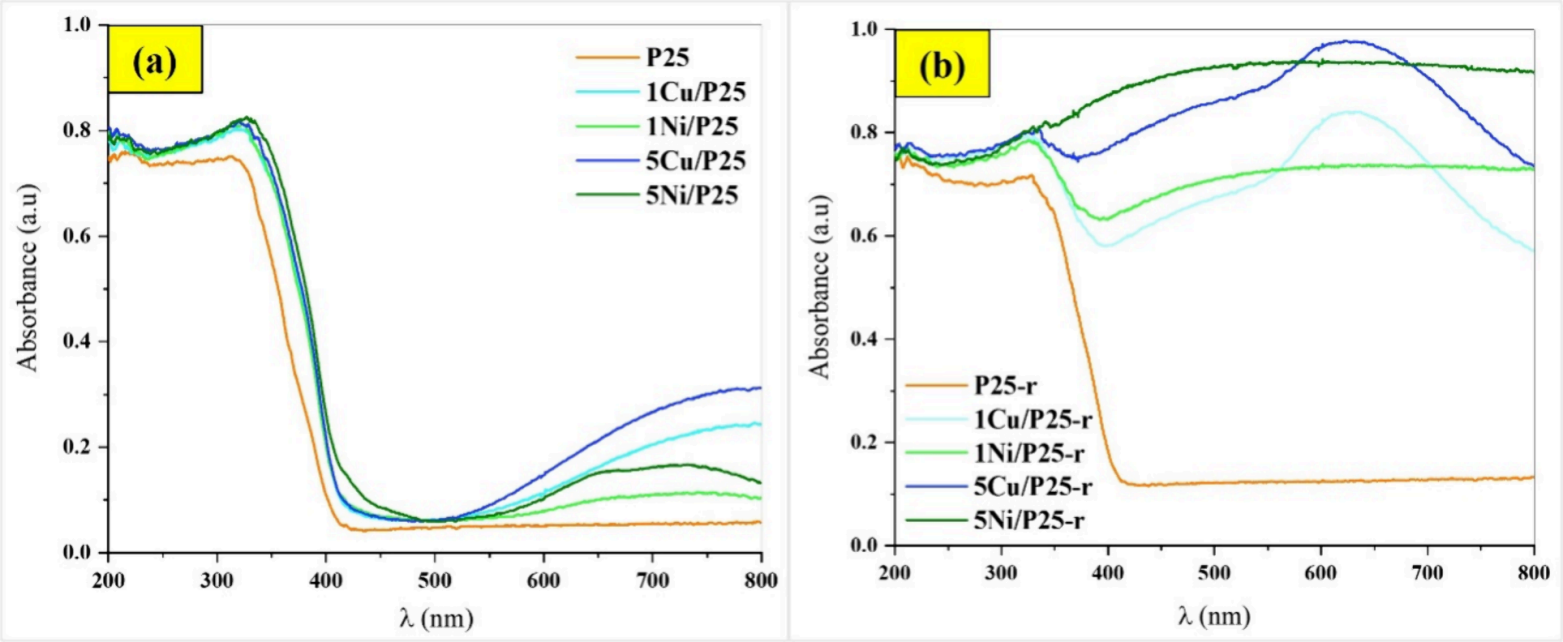
UV-vis diffuse reflectance absorption spectra for the photocatalysts: (a) XM/P25, and (b) XM/P25-r, compared with P25 and P25-r, respectively.

The increased absorbance observed in the XM/P25 photocatalysts is attributed to electronic transitions associated with the loaded metal species. Specifically, the absorbance below 400 nm is ascribed to ligand-to-metal charge transfer (LMCT) transitions from oxygen anions (O^2–^) to Ti^4+^ and metal species, consistent with findings reported in the literature.^42^ In contrast, the absorbance above 500 nm is primarily attributed to d-d transitions within the metal species, with Cu^2+^ (3d^9^ configuration)^42^ and Ni^2+^ (3d^8^ configuration)^43^ being the main contributors to these transitions.

The absorbance for P25-r in the 200–350 nm range is slightly lower than for P25, but it surpasses that for P25 in the visible range, rising notably (0.10 vs 0.05) from around 400 nm onward. The characterization data (from XRD, N_2_ adsorption–desorption, XPS, and TG) has shown that hydrogen reduction mainly alters the surface properties of P25, affecting its optical behavior while preserving its bulk structure. The lower UV absorbance of P25-r is supposed to be the result of a slightly reduced surface area and fewer surface hydroxyl groups content, as confirmed by N_2_ adsorption–desorption and TG, respectively. However, the increased visible absorbance is likely due to surface sensitization during the thermal reduction treatment.^44^

The absorbance for the XM/P25-r photocatalysts is similar to that for XM/P25 in the lower λ range but much higher from 350 nm onward, indicating substantial changes induced by the thermal reduction treatment, in line with published data.^45^ These differences at higher wavelengths reflect the impact of the treatment on the surface properties and electronic structure of the materials.

When comparing the effect of the metal, notable differences emerge: the absorbance of 1Cu/P25-r and 5Cu/P25-r, with bluish-gray and slightly darker bluish-gray colors, respectively (Figure S2), imply a plasmon effect at about 650 nm, attributed to metallic copper species.^46^ In contrast, the absorbance of 1Ni/P25-r and 5Ni/P25-r, gray and darker gray (almost black) colors, respectively (see Figure S2), remain more uniform along the entire visible spectrum, confirming the presence of supported metallic nickel nanoparticles.^43^ Hence, in addition to XRD and XPS, UV–vis analysis further supports the formation of the zero-valent states upon thermal reduction. Furthermore, the absorbance in the visible range is higher for the 5M/P25-r samples compared to the 1M/P25-r samples, irrespective of the metal used, consistent with the metal content.

Hence, in addition to XRD and XPS analysis, the UV-vis absorption spectra of XCu/P25 and XCu/P25-r photocatalysts can be helpful in identifying the Cu species present. The (as-prepared) XCu/P25 photocatalysts show broad absorption features above 500 nm without a distinct localized surface plasmon resonance (LSPR) peak which suggests, as mentioned before, the presence of Cu in oxidized states. After thermal reduction (XCu/P25-r), the photocatalysts display a pronounced broad absorbance peak between 400–550 nm, as well as a distinct peak in the visible range around 560–760 nm. The first peak is attributed to factors including the LSPR of Cu^0^ nanoparticles, d–s transitions in Cu^0^, interfacial charge transfer from TiO_2_ to Cu^2+^, and metal-to-ligand charge transfer (MLCT) associated with Cu^+^.^42^ The second peak is specifically linked to the LSPR of Cu^0^ nanoparticles.^46^

The band gap values calculated by the Tauc plot using the Kubelka–Munk method are presented in Table 2. It was not possible to determine the *E*_g_ for 5Cu/P25-r and 5Ni/P25-r due to the strong visible absorbance.

**Table 2:** Calculated Band Gap Energies for the Studied Photocatalysts and Their Conversion to the Corresponding Wavelengths (λ)

Photocatalyst	*E*_g_ (eV)	λ (nm)
P25	3.01	412
1Cu/P25	2.92	425
1Ni/P25	2.91	426
5Cu/P25	2.88	430
5Ni/P25	2.90	428
P25-r	2.95	420
1Cu/P25-r	2.33	532
1Ni/P25-r	1.97	629
5Cu/P25-r	not determined	>600
5Ni/P25-r	not determined	>600

The reference photocatalyst P25 exhibits a band gap value of 3.01 eV, perfectly in line with the values reported in the literature.^47^ The other photocatalysts present lower band gap energies, with P25-r, 1Cu/P25, 1Ni/P25, 5Cu/P25, and 5Ni/P25 all displaying close values ranging from 2.88 to 2.95 eV, whereas noticeably lower values are observed for the 1M/P25-r photocatalysts. Although the *E*_g_ for 5M/P25-r could not be reliably determined using the Kubelka–Munk method due to their significant absorbance in the visible light range, the shift in the onset edge of absorbance toward higher wavelengths for both photocatalysts suggests a lower band gap.

The recorded PL spectra for all the studied photocatalysts are presented in Figure 6. These results provide direct evidence of the electron–hole pairs recombination rates in the photocatalysts, emphasizing the effects of the metal, its concentration, and the reduction treatment. As shown in Figure 6a, all the XM/P25 photocatalysts exhibit lower PL emission peak intensities than P25, indicating a decrease in the electron–hole pairs recombination rates.^48^ Notably, the incorporation of Cu species results in a more pronounced reduction in the emission peak intensity compared to Ni ones. For both metals, increasing the metal content from 1% to 5 wt % significantly enhances the electron–hole pairs separation efficiency. Consequently, the order of electron–hole pairs separation efficiency is as follows:

5Cu/P25>1Cu/P25>5Ni/P25>1Ni/P25>P25

**Figure 6:**
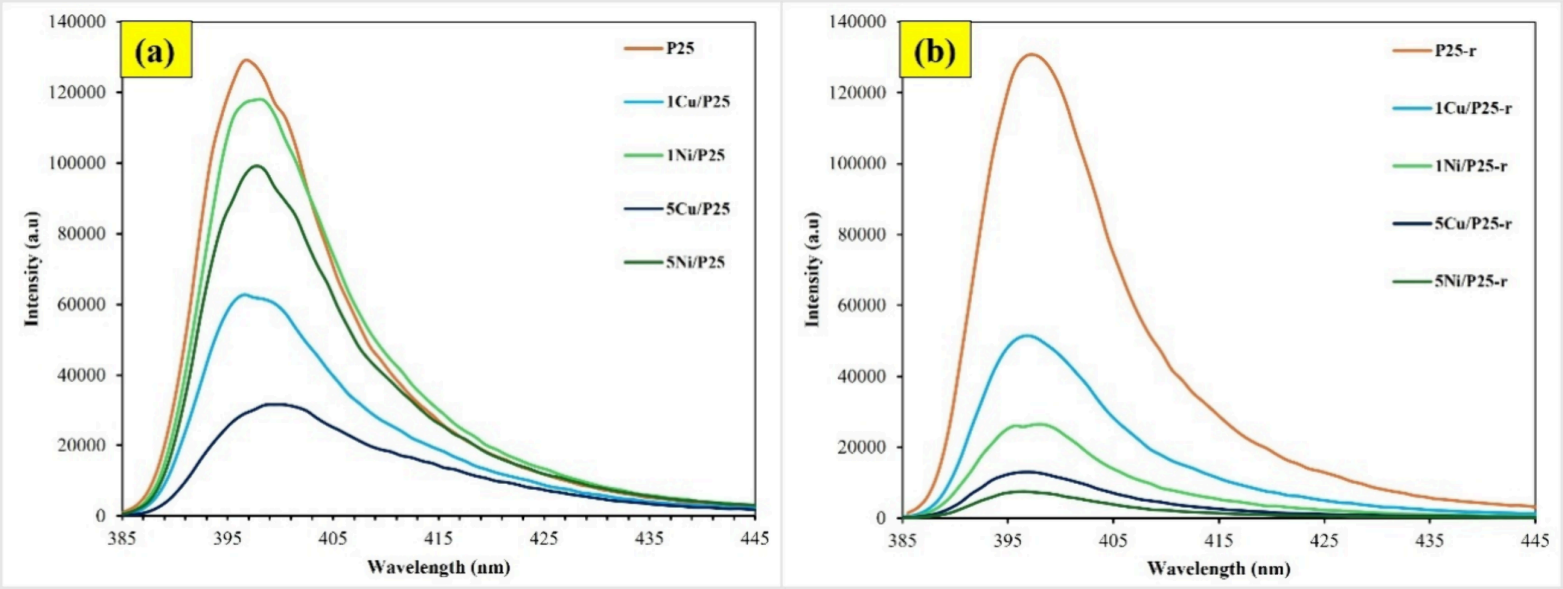
Photoluminescence spectra measured after excitation of the photocatalysts at λ = 373.5 nm for: (a) P25 and XM/P25, and (b) P25-r and XM/P25-r photocatalysts.

A similar overall PL profile is observed for P25 and P25-r (Figure 6a, b, respectively). In the XM/P25-r series (Figures 6b), lower PL emission peak intensities are observed compared with XM/P25, with reduced Ni being more effective than reduced Cu in decreasing the electron–hole pairs recombination rate. Again, 5 wt % metal is more efficient than 1 wt %. Accordingly, the order of electron–hole pairs separation efficiency for the thermally reduced photocatalysts is as follows:

5Ni/P25‐r>5Cu/P25‐r>1Ni/P25‐r>1Cu/P25‐r>P25‐r

Combining the results from both series (see Figure S9 in the Supporting Information), the overall order of electron–hole pairs separation efficiency is as follows:

5Ni/P25‐r>5Cu/P25‐r>1Ni/P25‐r>5Cu/P25>1Cu/P25‐r>1Cu/P25>5Ni/P25>1Ni/P25>P25‐r≈P25

Overall, the PL spectra reveal that metal incorporation, especially when followed by thermal reduction, effectively reduces the electron–hole pairs recombination in TiO_2_-based photocatalysts, with Ni^0^ outperforming Cu^0^. These modifications are promising strategies for enhancing the photocatalytic performance.

In summary, the characterization of the photocatalysts, particularly XM/P25 and XM/P25-r, reveals critical physicochemical, optical, and electronic features. These include the distribution of metal species, their oxidation states, and their interaction with the TiO_2_ support. These insights provide better understanding of the effects of metal incorporation, quantity, and thermal reduction, which are essential for optimizing their performance in WS.

Specific findings from XRD, XPS, and TEM-EDS analyses show that for XM/P25, produced by impregnation followed by low-temperature drying at 80 °C, the metal species are primarily located on the TiO_2_ surface rather than being incorporated into the lattice. This suggests that the observed effects arise from metal loading or heterojunction formation rather than lattice doping.

Although XRD could not detect distinct metal-related components, XPS and UV–vis DRS analyses revealed that Cu and Ni are likely present as oxides or hydroxides, with some metal nitrate species (e.g., Cu_2_O, Cu­(OH)_2_, Cu­(NO_3_)_2_ for Cu-containing photocatalysts, and Ni­(OH)_2_ and Ni­(NO_3_)_2_ for Ni-containing ones). TEM and TEM-EDS confirmed the formation of nanometric metal species, with Cu species typically appearing as nanoparticles smaller than 3 nm, while Ni species were usually smaller, making precise size determination challenging. UV–vis DRS analysis showed slight shifts in the band gap energies of Cu- and Ni-modified P25 photocatalysts compared to P25 TiO_2_, with the band gap values decreasing from 3.01 eV for P25 to around 2.88–2.91 eV for the modified materials. This indicates a modest extension of light absorption into the visible region, potentially improving the photocatalytic activity under UV–visible light. Additionally, PL spectra revealed reduced electron–hole pairs recombination rates, with Cu outperforming Ni and 5% metal loading being more effective than 1%. An increased metal content provides additional electron-trapping sites, reducing recombination, particularly in Cu-containing photocatalysts, which highlights enhanced photogenerated charge carrier separation.

After thermal reduction at 500 °C, XRD, XPS, and UV–vis DRS analyses of the XM/P25-r series confirmed the formation of Cu^0^ and Ni^0^, often coexisting with oxides such as CuO and NiO. TEM and TEM-EDS showed these materials as nanometric domains and larger clusters (>30 nm). Despite the high-temperature treatment, these metallic species remain confined to the surface of TiO_2_ without integrating into the lattice, primarily affecting the surface properties. The reduction process also led to a significant decrease in the band gap, with values as low as 2.33 eV for 1Cu/P25-r or 1.97 eV for 1Ni/P25-r. This narrowing of the band gap allows for greater absorption in the visible region, extending beyond 600 nm for some materials, thereby enhancing the photocatalysts’ ability to utilize a broader spectrum of light. In copper-containing photocatalysts, the formation of metallic Cu^0^ induces surface plasmon resonance, which intensifies the local electromagnetic field around the particles, increasing light absorption. LSPR can generate “hot electrons” from Cu that inject into the TiO_2_ conduction band, generating additional electron–hole pairs and improving charge carrier dynamics. PL spectra indicated further enhanced charge carrier separation, evidenced by even lower electron–hole pairs recombination rates than in the as-prepared photocatalysts.

### 3.2 Photocatalyzed Water Splitting Results

Figure 7 plots the accumulated hydrogen generation (for 5 h irradiation experiments) as a function of time determined for the tests that resulted in H_2_ generation, and Table 3 compiles the amount of hydrogen produced in each test. These data have been obtained from the continuous hydrogen generation profiles shown in Figure S10 in the Supporting Information. Additionally, the hydrogen production rate (μmol h^–1^) is displayed in Figure S11 in the Supporting Information. It is important to note that hydrogen was not detected in the tests conducted with the as-prepared (XM/P25) photocatalysts.

**Figure 7:**
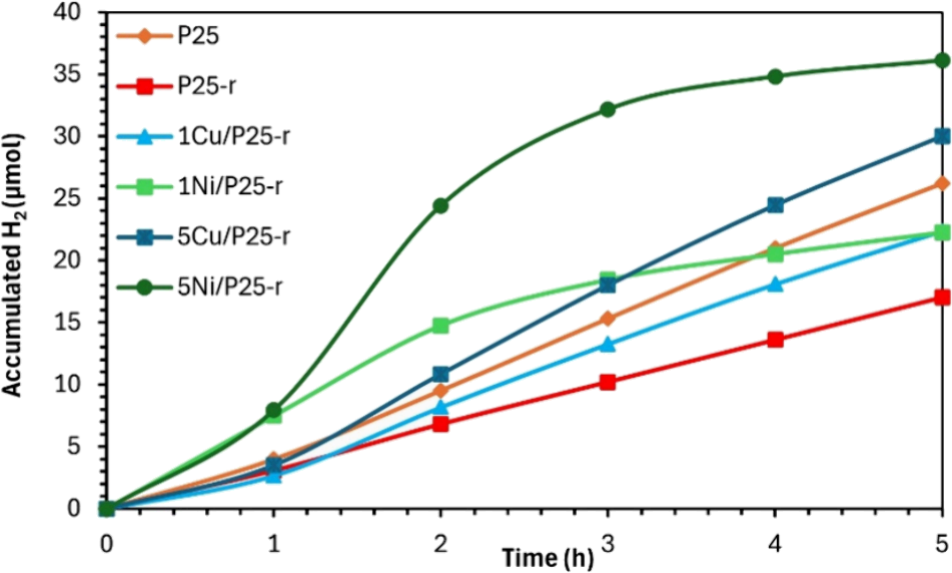
Accumulated hydrogen generation vs time in the UV photocatalyzed water splitting experiments using P25, P25-r, and XM/P25-r photocatalysts.

**Table 3:** Amount of H_2_ Generated by Water Splitting Using P25 as Well as XM/P25, P25-r, and XM/P25-r, Expressed in Total μmol of H_2_ after 5 h Reaction Time and as μmol·h^–1^·g_cat_^–1^

Test #	Photocatalyst	H_2_ (μmol)	H_2_ (μmol·h^–1^·g_cat_^–1^)
1	-	-	-
2	P25	26.1	261
3	P25-r	15.7	157
4	1Cu/P25	-	-
5	1Cu/P25-r	19.8	198
6	1Ni/P25	-	-
7	1Ni/P25-r	21.3	213
8	5Cu/P25	-	-
9	5Cu/P25-r	28.4	284
10	5Ni/P25	-	-
11	5Ni/P25-r	33.1	331

The blank experiment (test #1), conducted without any photocatalyst, did not show hydrogen formation. This serves as a baseline for evaluating the catalytic activity of the modified and unmodified photocatalysts.

In the photocatalyzed tests (tests #2 to #11), the reference photocatalyst P25 (test #2) generated some hydrogen, confirming its ability to catalyze WS under the studied conditions. In contrast, the as-prepared metal-modified photocatalysts (XM/P25) did not produce any hydrogen. After the thermal reduction treatment, P25-r produced less hydrogen than the untreated P25, indicating a loss of photocatalytic activity. Interestingly, the metal-modified photocatalysts that underwent thermal reduction (XM/P25-r) exhibited hydrogen generation, unlike their as-prepared counterparts. All the XM/P25-r photocatalysts outperformed P25-r, though they were only slightly better or within the range of P25.

The distinct outcomes in hydrogen production among the photocatalysts highlight the crucial role of incorporating metal species and the impact of the thermal reduction treatment.

The absence of hydrogen production when using the XM/P25 photocatalysts can be attributed to the deactivation of TiO_2_ by the deposited metal species. XRD, N_2_ adsorption–desorption, and TEM did not provide direct insights about the lack of hydrogen generation in this case, although they remarked that these non-reduced materials involve a high metal species coverage of the P25 surface (XPS data reveal a high surface metal content, and TEM data a high dispersion of these species). XPS and PL analysis offered valuable explanations. XPS revealed a slight reduction in the content of surface −OH groups, decreasing from 10% in P25 to 6–7% in the metal-loaded samples (XM/P25). This reduction does not necessarily indicate a loss of total surface oxygen but suggests a redistribution. The deposition of metal may partially mask the original −OH groups while introducing other oxygen-containing species, such as adsorbed water or hydrated compounds. This change is evident in the O 1s spectra. These new oxygen species, combined with elevated O/Ti ratios observed in the metal-loaded samples, suggest that the altered surface chemistry may obscure or modify the active sites crucial for hydrogen production, thereby reducing the photocatalytic activity of TiO_2_. Additionally, XPS analysis showed that the metal species on the photocatalysts are predominantly in their oxidized forms, copper as Cu^+^ and Cu^2+^, and nickel as Ni^2+^, and a negligible proportion of metallic (zero-valent) active sites, which are those with a more relevant role decreasing the electron–hole pairs recombination rate. This suggests that photogenerated electrons from UV excitation are likely being consumed to reduce the metal species rather than directly contributing to hydrogen production. If this is the case, the deposited metal species, instead of enhancing the photocatalytic activity, are detrimental, as they consume the photogenerated electrons and may also hinder the exposure of active sites by potentially covering parts of the TiO_2_ surface. PL data further indicated that incorporating metal species significantly enhances charge separation. Although no H_2_ is formed, this supports the explanation based on the consumption of the photogenerated electrons for the reduction of the oxidized metal species. The combination of covering the TiO_2_ surface and consuming photogenerated electrons to reduce the metal species may explain the observed quenched photocatalytic activity of TiO_2_ after metal incorporation in XM/P25, resulting in no hydrogen formation.

To investigate further, an extended experiment (15 h of irradiation) was conducted using the 5Cu/P25 photocatalyst (further details are provided in the Experimental Section). The corresponding data are presented in Figure S12 and Table S5, both in the Supporting Information. The results revealed negligible hydrogen generation during the first 10 h of UV irradiation. However, from 10 to 15 h, a notable hydrogen production was observed. This generation occurred after a color change in the 5Cu/P25 photocatalyst, from light blue to dark bluish gray, which also was observed after 10 h of irradiation. Specifically, the color of the 5Cu/P25 photocatalyst after in situ reduction closely resembled that of 5Cu/P25-r, reduced under a controlled hydrogen atmosphere (see Figure S13 in the Supporting Information). This similarity suggests that the in situ reduction under photocatalytic conditions occurs, inducing changes in the metal oxidation state of the co-catalyst similar to those achieved with an H_2_ atmosphere reduction treatment, where a confirmed copper reduction process took place. Although in situ characterization was not possible due to equipment limitations and considering the risk of reoxidation during air exposure after the reaction, the notable change in the appearance of the photocatalysts provides indirect evidence of the in situ reduction of Cu species that occurred during the WS process.

These results support the hypothesis that photogenerated electrons are consumed in reducing metal species. Even if the metal species are in situ reduced, as it has been proved with a Cu-containing catalyst in a 15 h irradiation experiment, it takes time to have the copper species reduced and to have a suitable activity, also noting that this reduction process can consume the electrons that could be generated on the P25 surface, necessary to produce hydrogen, so H_2_ would not be generated in the process until the zero-valent metal appears/prevails. This is not the only mechanism that would explain the in situ metallic species reduction. According to the literature, UV photons from a low-pressure mercury lamp can slowly reduce both CuO and Cu_2_O at room temperature,^49^ and this reduction would be favored for highly dispersed particles.

Furthermore, the obtained results indicate that XM/P25-r photocatalysts, where metals are reduced and contain at least a portion of their zero-valent form, are photocatalytically active and can produce measurable hydrogen during water splitting.

The decreased activity of P25-r compared to P25 can be primarily attributed to chemical modifications induced by the thermal treatment, with minimal contributions from structural changes. A substantial reduction in surface −OH groups, which are critical for photocatalytic activity, has occurred in P25-r.^50,51^ Additionally, the slight porosity reduction may also contribute to the decreased performance by lowering the number of active sites available for reactions.^52^

Thermal reduction treatment typically reinforces metal–support interactions,^53,54^ an evident phenomenon in multiple analytical results for the XM/P25-r photocatalysts. XRD revealed the formation of metallic phases in reduced samples, while XPS data showed changes in the oxidation states, including some Ti^3+^ formation (observed in 5Cu/P25-r) and reduction of metals to their zero-valent states, with consequences in the UV–vis DRS data. Additionally, shifts in the metal oxidation states and XPS positions suggest SMSI, improving the electron transfer, as confirmed by PL and, consequently, the photocatalytic activity.

Furthermore, combining the information provided by UV–vis DRS spectra (Figure 6 and Table 2), with the irradiation spectrum of the UV lamp used (Figure S1a in the Supporting Information), it can be concluded that the XM/P25-r photocatalysts can more efficiently utilize all the wavelength region of the light source, absorbing and generating electron–hole pairs at wavelengths that are unsuitable for their XM/P25 counterparts.

The improved photocatalytic activity of XM/P25-r under UV light compared to P25 can be primarily attributed to the presence of metals in zero oxidation states. The plasmon resonance of Cu metallic species, along with the formation of oxygen vacancies and Ti^3+^ sites, plays a key role. These effects improve charge carrier dynamics, with reduced metal species acting as electron sinks that capture photogenerated electrons from the TiO_2_ conduction band. Additionally, metal species provide catalytic sites that facilitate faster electron transfer to protons, aiding in the reduction of protons to hydrogen at the metal–semiconductor interface. The plasmon resonance enhances light absorption and also generates electrons, contributing to improved charge separation and photocatalytic efficiency.^55^ Furthermore, oxygen vacancies and Ti^3+^ sites in 5Cu/P25-r specifically improve electron mobility and prolong charge separation times.^56^ Together, these factors significantly enhance photocatalytic H_2_ evolution compared to XM/P25. The improved interactions between the metal and the TiO_2_ support, combined with a more efficient utilization of the light source, as evidenced by UV–vis data, further contribute to this improvement. Notably, the best results are observed for X = 5 wt %. The lower activity observed for 1M/P25-r compared to P25 can be explained by a balance between the activity enhancement due to the presence of reduced metal species, deduced by XRD, XPS, Auger spectroscopy and further confirmed by UV–vis/DRS, and the offset by the decrease in −OH active sites content on the TiO_2_ surface after the reduction treatment (as deduced by XPS and specifically TG over P25 and P25-r). In any case, the optimum metal content to maximize the efficiency could be determined in future studies, keeping in mind that it would depend on the metal chosen and the experimental preparation conditions.

Although the band gap of P25 is significantly larger than that of the XM/P25-r photocatalysts, its comparable or superior performance may be attributed to its maximum wavelength absorbance, which closely aligns with one of the primary irradiation peaks of the lamp (∼405 nm), enabling optimal light absorption. Additionally, while XM/P25-r benefits from enhanced charge separation due to metal modification, several factors might limit their overall performance. The rate-limiting step in hydrogen production could be surface reactions^57,58^ or mass transfer^59,60^ rather than charge separation^4^. Furthermore, there may be possible electron and hole pairs recombination at metal sites before H_2_ formation occurs, reducing the efficiency.^61^

The presence of metal species on the TiO_2_ surface can also block active sites, particularly at higher loadings, impeding the reaction.^61^ Moreover, the metal agglomeration at higher loadings creates larger metallic domains that are less effective for H_2_ evolution.^62,63^ These combined factors might explain why P25 achieves better overall photocatalytic performance despite its larger band gap.

Thus, these results suggest that incorporating Cu or Ni into P25 enhances hydrogen generation if the metal is, at least partially, reduced to the zero-valent state, and a minimum metal content, likely above 1% and closer to 5 wt %, is present.

Comparing the impact of Cu and Ni on the activity of the photocatalysts (in the case of XM/P25-r), it is evident that although both result in comparable total hydrogen production, with XNi/P25-r showing slightly higher yield than XCu/P25-r, their kinetics of H_2_ evolution differ significantly. These differences are illustrated in Figure 8 and Figures S10 and S11 of the Supporting Information, with Figure S11 particularly highlighting the H_2_ evolution rate.

**Figure 8:**
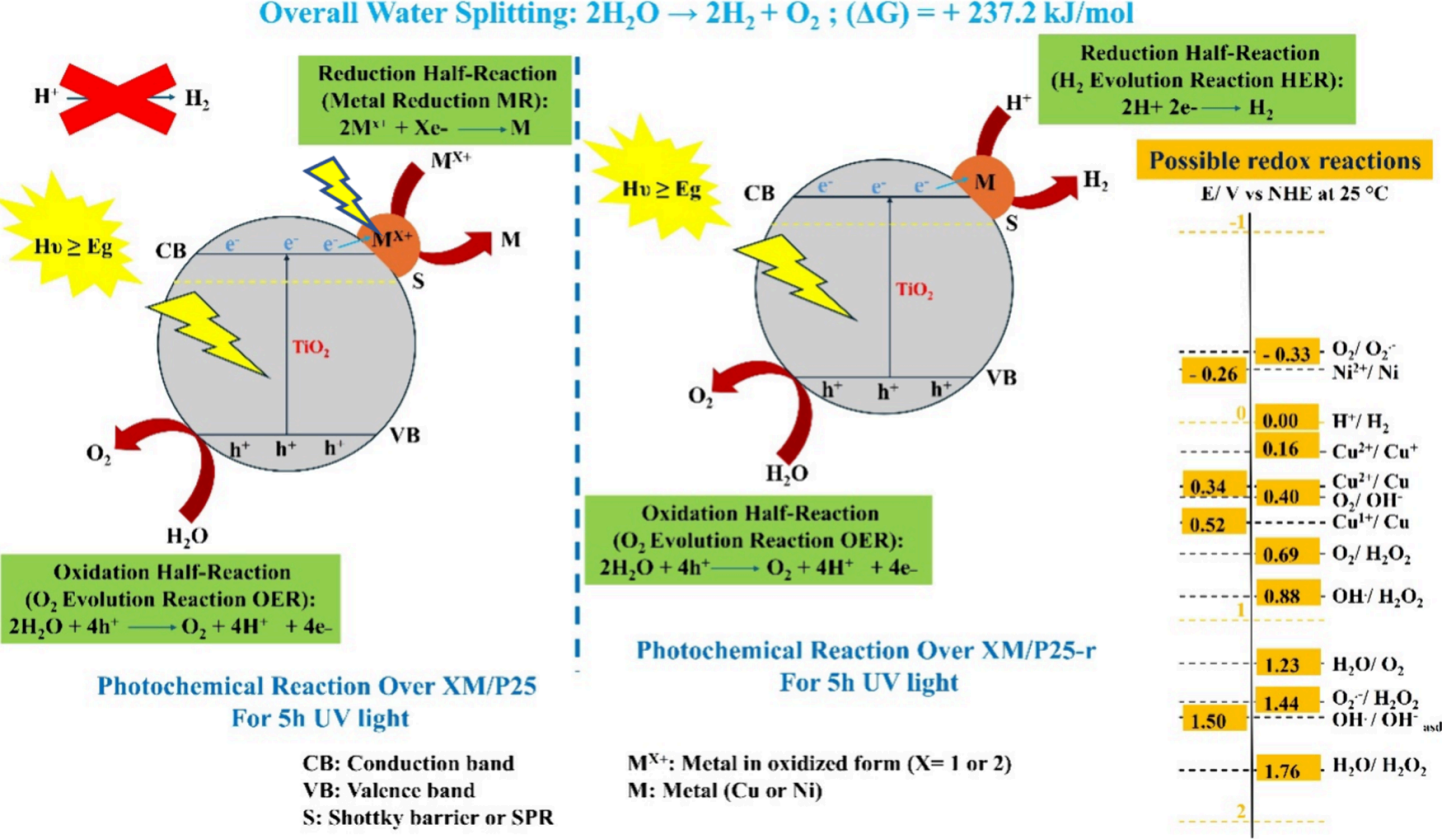
Proposed redox mechanism during photochemical WS tests over XM/P25 and XM/P25-r photocatalysts: electrons and/or UV light photons consumption for metal reduction on XM/P25 (WS is not detected) and hydrogen production on XM/P25-r (WS occurs).

To evaluate the early hydrogen production rate, the initial slope of each curve in Figure 7 (from 0 to 1 h) was calculated. The XNi/P25-r photocatalysts exhibit a steeper initial slope compared to XCu/P25-r, indicating a rapid onset of hydrogen generation. This is consistent with the data in Figures S10 and S11, where Ni-containing photocatalysts reach peak hydrogen concentrations of approximately 129 and 164 ppm and rates of 7.17 and 7.28 μmol h^–1^ for 1Ni/P25-r and 5Ni/P25-r, respectively, within just 1 h of reaction time, highlighting their fast initial kinetics. In contrast, XCu/P25-r photocatalysts display a more gradual hydrogen production increase. Specifically, 1Cu/P25-r reaches a peak of approximately 70 ppm with a rate of 2.36 μmol h^–1^, while 5Cu/P25-r achieves around 94 ppm with a rate of 3.32 μmol h^–1^, both at approximately 1 h of reaction time, reflecting steadier kinetics. After reaching a rapid maximum peak, approximately 139 and 167 ppm at 0.80 and 1.29 h for 1Ni/P25-r and 5Ni/P25-r, respectively, XNi/P25-r photocatalysts experience a sharp decline in hydrogen production rates, suggesting a rapid decrease in catalytic activity. This decline reflects the deactivation of Ni, attributed to the oxidation of Ni from Ni^0^ to Ni^2+^. This oxidation may occur directly through interaction with valence band holes or via hydroxyl radicals (OH·) generated during UV irradiation^16^ or by the O_2_ produced during water splitting.^64^ The deactivation of Ni-containing photocatalysts results from Ni’s limited redox flexibility. In these photocatalysts, Ni exists as Ni^2+^ and Ni^0^. During the reaction, while the oxidation of Ni^0^ to Ni^2+^ is thermodynamically favorable and easily occurs, facilitated, for instance, by the photogenerated holes, as described earlier, the reverse reduction process (Ni^2+^ to Ni^0^) is more challenging. This is due to the potential of −0.23 V vs SHE, which is only slightly less negative than the one for the conduction band in TiO_2_, with potential around −0.5 V vs SHE at pH 7. This limited reduction capacity restricts Ni’s ability to undergo rapid, repeated cycles of oxidation and reduction, thereby reducing its ability to regenerate active sites and sustain long-term catalytic activity.^65^

In contrast, XCu/P25-r photocatalysts exhibit a slower decline in hydrogen production, indicating more stable kinetics. The more gradual decrease in the production rate after the maximum peak suggests sustained activity over time compared to Ni-containing photocatalysts. This behavior is explained by Cu’s unique redox behavior during the photocatalytic process. During water splitting, photogenerated holes can oxidize Cu^0^ to Cu^+^ or Cu^2+^, even in the absence of atmospheric oxygen, as the reaction generates oxidizing species such as OH· or directly photogenerated holes. Meanwhile, oxidized Cu (Cu^+^, Cu^2+^) can be easily reduced to Cu^0^ by the photogenerated electrons.^65^ Hence, Cu’s ability to stably exist in multiple oxidation states (Cu^0^, Cu^+^, Cu^2+^) facilitates redox cycling without catalyst deactivation.^66^ The localized oxidizing environment created by oxygen produced during water splitting further supports Cu-based photocatalysts’ sustained activity. Together, these factors explain the prolonged and reliable performance of Cu-containing photocatalysts over extended reaction times.

Thus, while thermally reduced Ni-containing photocatalysts are capable of rapid hydrogen generation, their swift decline after peaking indicates less stable performance. In contrast, copper-containing photocatalysts demonstrate a gradual yet sustained performance.

Water splitting produces hydrogen and oxygen as products.^2^ In the performed experiments, although both gases were detected, only H_2_ was quantified. The O_2_ signal (*m*/*z* = 32) shown in Figure S14 of the Supporting Information, reveals that O_2_ evolution does not follow the same trend as H_2_ production. This can be attributed to several factors. One factor is the difference in solubility between the two gases, with O_2_ being more soluble in water than H_2_ under similar conditions.^67^ Consequently, some O_2_ may remain dissolved in the aqueous phase, delaying its detection as a gas. Furthermore, O_2_ can be consumed in competing reactions, such as the oxidation of reduced metal species on the TiO_2_ surfaces^64^ or reduction processes leading to the formation of hydrogen peroxide (H_2_O_2_),^68^ thereby decreasing the observed O_2_ evolution. H_2_O_2_ formation is another contributing factor. Partial oxidation of water by photogenerated holes can produce H_2_O_2_, diverting charge carriers and reducing O_2_ production.^68,69^ Reactive oxygen species, i.e., O_2_·^–^ and OH· also contribute to H_2_O_2_ formation.^68,69^ Under photocatalytic conditions, H_2_O_2_ is unstable and decomposes into water and O_2_ over time, which may delay the observable O_2_ evolution relative to H_2_.^69^ The observed O_2_ signals further highlight the effect of material modifications and reaction conditions. For instance, P25 shows a slow and weak O_2_ signal over time (Figure S14a), whereas P25-r exhibits more pronounced O_2_ evolution (Figure S14b), indicating a higher O_2_ production after thermal reduction. Metal-modified photocatalysts also display distinct O_2_ evolution behaviors. 1Cu/P25-r shows a stable but lower O_2_ signal compared to 5Cu/P25-r, which exhibits a steady upward trend, reflecting consistent O_2_ generation. In contrast, Ni-modified photocatalysts demonstrate different dynamics: 1Ni/P25-r produces a weak and delayed O_2_ signal detectable only after 4 h of reaction, while 5Ni/P25-r generates a stronger and more sustained O_2_ signal.

Given the good optical properties of XM/P25-r photocatalysts, their performance under visible light was evaluated, with 5Cu/P25-r selected as the representative material. The results indicate that, despite the observed optical enhancement, no hydrogen production was detected after 5 h of irradiation, highlighting the limited efficiency of these photocatalysts under visible light. Also, note the much lower irradiance for the TXE150 lamp. Further investigation is needed to optimize the photocatalyst structure and enhance their activity under visible light.

### 3.3 Mechanism of the Photocatalyzed Water Splitting by Metal Modified-TiO_2_ Photocatalysts

Under UV light excitation, TiO_2_ photocatalysts absorb photon energy equal to or greater than their band gap, generating electrons in the conduction band (CB) and holes in the valence band (VB). For an efficient photocatalytic activity, the photogenerated electrons and holes must successfully migrate to the surface of the catalyst to participate in redox reactions. Electrons in the CB can transfer to metal particles such as Cu or Ni species, which have a lower work function than TiO_2_. These metal species act as electron sinks, effectively capturing electrons and preventing their recombination with holes. This separation ensures that the photogenerated holes remain available to drive the water oxidation reaction, resulting in the formation of O_2_. Simultaneously, the captured electrons reduce protons at the metal-TiO_2_ interface, leading to the production of H_2_.^54^ In this scenario, it is important that some reduced metal exists. Hydrogen generation did not occur for the as-prepared photocatalysts, attributed to the photogenerated electrons being consumed in the reduction of metal species, delaying the hydrogen formation, which does not occur until the in situ reduction of the metal species occurs (confirmed by a UV light experiment over 5Cu/P25, where hydrogen was only detected after 10 h of irradiation). Note that, according to the literature, UV photons from a low-pressure mercury lamp can slowly reduce both CuO and Cu_2_O at room temperature,^49^ being such reduction easier for highly dispersed particles.

In contrast, the photocatalytic activity is greatly enhanced in the presence of Cu^0^ or Ni^0^, which act as efficient electron traps on TiO_2_, capturing photogenerated electrons from the TiO_2_ conduction band and directly using them for the reduction of protons to form hydrogen, as confirmed by the photocatalytic results. The improved charge separation is evidenced by a marked reduction in the PL intensity. The formation of Cu^0^ induces Surface Plasmon Resonance, which enhances the local electromagnetic field around the particles. LSPR generates ″hot electrons″ that inject into the TiO_2_ conduction band, further improving charge carrier dynamics.

Photocatalyzed WS produces H_2_ and O_2_, but side reactions and/or the modification of the metal species can occur, reducing the efficiency. Thus, the excited electrons may reduce O_2_, creating reactive oxygen species (ROS) like O_2_·^–^ and OH·, which can lead to photocatalyst oxidation, charge recombination, or hydrogen peroxide formation. These side reactions compete with the hydrogen evolution reaction (HER), lowering hydrogen generation efficiency. Holes in the valence band and the oxygen generated in the WS can also oxidize the metal species, reducing charge carriers for the oxygen evolution reaction (OER) and decreasing the efficiency of the process. Additionally, H_2_ and O_2_ gases may recombine on the catalyst surface, especially with metal co-catalysts, further reducing overall gas production.

Figure 8 illustrates the proposed redox mechanism during photochemical water splitting tests over XM/P25 and XM/P25-r photocatalysts, highlighting the electrons’ and/or photons consumption for metal reduction on XM/P25 (WS does not occur), and hydrogen production on XM/P25-r (WS occurs). In the XM/P25 photocatalysts, once Cu^0^ or Ni^0^ are present, the photocatalyzed water splitting process would start.

In the context of hydrogen generation via photocatalytic water splitting, comparison with previously published data may allow a better evaluation of the activity and performance of the studied metal/TiO_2_ photocatalysts. Such a comparison is challenging since almost all the published works involve the H_2_ production by TiO_2_-photocatalysed water splitting in the presence of liquid and very reactive sacrificial agents (i.e., methanol, ethanol, glycerol, etc.) aiming to enhance the process efficiency. Some of the previously published data on TiO_2_-photocatalyzed water splitting are summarized in Table S6 in the Supporting Information.

Upon comparing our results with those few data reported in the literature in Table S6, consistent observations emerge: (i) TiO_2_ photocatalysts show low photocatalytic activity for the water splitting process, (ii) an improvement in the activity is observed when using UV light over visible light, (iii) the metal-containing TiO_2_ photocatalysts without reduction treatment or adequate calcination at higher temperatures fail to exhibit a measurable photocatalytic activity and (iv) despite the enhancement achieved through metal TiO_2_ modification (with metals reduction treatment), the hydrogen yield remains relatively low.

## 4 Conclusions

This study investigates the synthesis and characterization of metal-modified TiO_2_ photocatalysts, focusing on Cu or Ni loading, their contents, and the impact of thermal reduction on photocatalytic hydrogen production via water splitting without any sacrificial agent.

The study of the XM/P25 photocatalysts revealed that the metal species, primarily located on the TiO_2_ surface as oxidized forms (Cu_2_O, Cu­(OH)_2_, Cu­(NO_3_)_2_, Ni­(OH)_2_, and Ni­(NO_3_)_2_), were well dispersed in nanometric domains. This led to a minimal reduction in the band gap of the photocatalysts. Photoluminescence data indicated improved suppression of charge recombination. However, these photocatalysts did not produce measurable hydrogen, likely due to the deactivation of active sites, such as surface hydroxyl groups, and particularly the consumption of photogenerated electrons by the metal species for their reduction.

Thermal reduction of these photocatalysts transformed the metal species into metallic Cu or Ni, alongside oxides, forming nanometric and clustered domains on the TiO_2_ surface. This process reduced the band gap and further enhanced the electron–hole pairs separation. These improvements led to measurable hydrogen production, confirming the importance of thermal reduction in optimizing the performance of the photocatalysts.

Among the tested photocatalysts, a 5% metal loading exhibited the best performance. The XM/P25-r photocatalysts produced higher hydrogen levels than P25-r but only showed modest improvements compared to P25. These results underscore the need for further optimization to maximize the efficiency of these photocatalysts.

Comparing Cu and Ni, both metals demonstrated similar total hydrogen production but their kinetics and stability varied significantly. Ni-modified photocatalysts enabled rapid hydrogen generation but suffered performance degradation over time. In contrast, Cu-modified photocatalysts showed more stable and prolonged activity, making them better suited for extended reactions.

This work demonstrates the critical role of non-noble metal species, their concentrations, and oxidation states in their photocatalytic performance. By omitting the conventional calcination step, the study introduces a more sustainable and energy-efficient approach to the preparation of photocatalysts and proves that in situ reduction of the metal species can take place, which implies some induction period before hydrogen is detected.

## Supplementary Material



## Notes

Appearance of the photocatalysts, irradiation profile of lamps (UV and visible), N_2_ adsorption–desorption isotherms, particles size distribution of the metal species, Ti 2p3/2 and O1s XPS spectra of photocatalysts, Auger spectra of Cu-TiO_2_ photocatalysts, TG analysis of P25 and P25-r photocatalysts, photoluminescence spectra of the studies photocatalysts, hydrogen evolution vs time and hydrogen evolution rate in the photocatalyzed water splitting tests, and O_2_ evolution in the photocatalyzed water splitting tests (PDF)
