# Supplementary material for: Evaluating H2 Production by Ultraviolet-Induced Water Splitting over (Cu or Ni)-TiO2 Nanoparticle Photocatalysts
Source: ACS Appl Nano Mater. 2025 Apr 17;8(17):8646–62. doi: 10.1021/acsanm.5c00100 (PMC12131184; doi:10.1021/acsanm.5c00100)
Supplement: Supplementary file 1 [file an5c00100_si_001.pdf]

Evaluating H_2 Production by Ultraviolet-Induced Water Splitting over (Cu or Ni)-TiO_2 Nanoparticle Photocatalysts

Supporting information

Meryem Bouchabou, Juan Manuel Rives López, María del Carmen Román Martínez, Maria Angeles Lillo-Rodenas*.

MCMA Group, Department of Inorganic Chemistry and Materials Institute (IUMA), Faculty of Sciences, University of Alicante, Ap. 99, E-03080 Alicante, Spain.

Telephone: + 34 965903545. E-mail: mlillo@ua.es.

1. Experimental

1.1. Light source characteristics

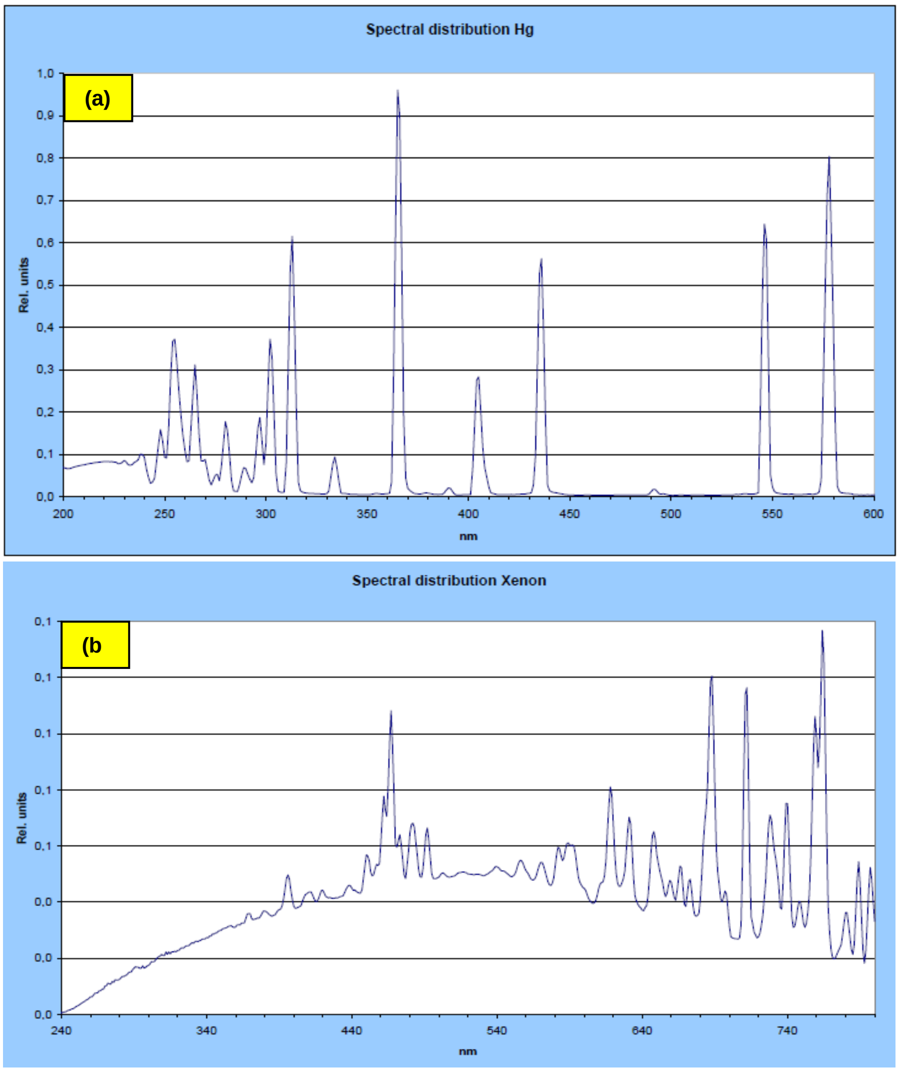

Figure S1. Irradiation profile of: a) TQ150 Z0 undoped UV lamp (150 W consumed, 47 W irradiated, λ_{max} = 365 nm) and b) TXE150 MK1 VIS lamp (150 W consumed, λ_{max} = 875 nm).

2. Results

2.1. Photocatalysts characterization

2.1.1. Appearance of the photocatalysts.

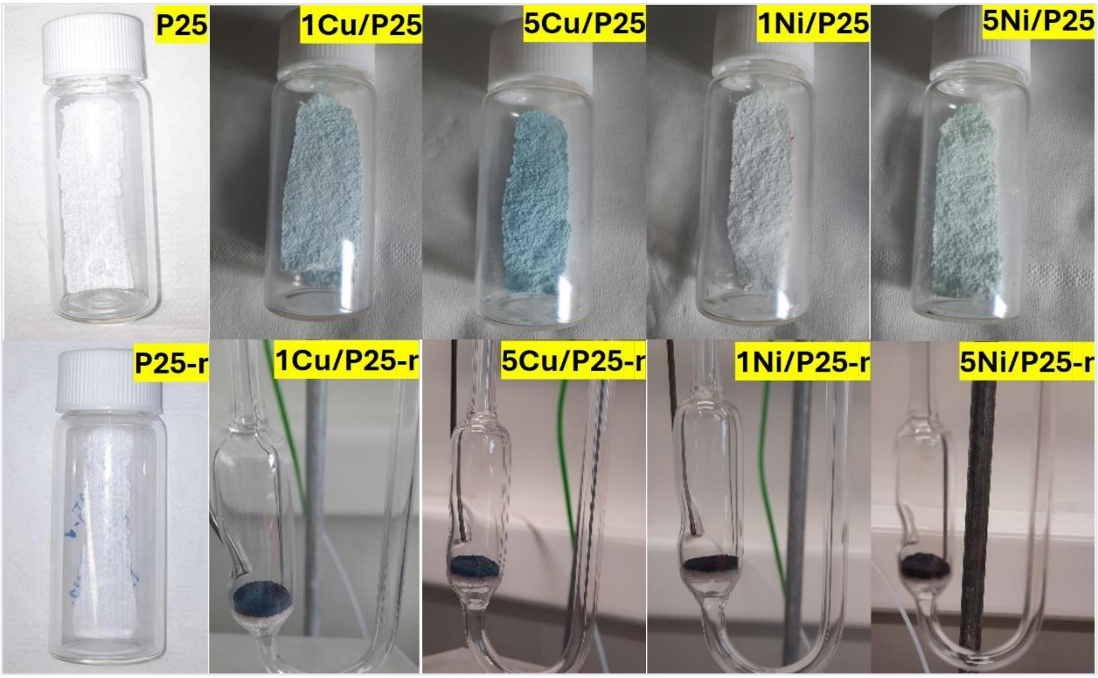

Figure S2. Photographs of all the studied photocatalysts.

2.1.2. N_2 adsorption-desorption

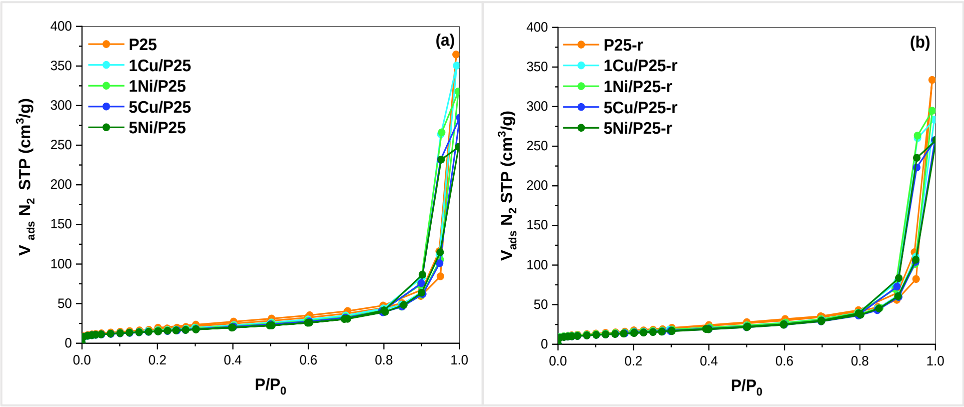

Figure S3. N_2 adsorption-desorption isotherms at -196 ºC for all the studied photocatalysts (a: XM/P25 and b: XM/P25-r).

Table S1. Textural properties for all the studied photocatalysts.

**Catalyst**	**BET (m^2/g)**	**V_{DR} N_2 (cm^3/g)**	**V_{meso} (cm^3/g)**	**V_{total} (cm^3/g)**
P25	68	0.02	0.06	0.56
1Cu/P25	60	0.02	0.08	0.54
1Ni/P25	57	0.02	0.07	0.49
5Cu/P25	55	0.02	0.07	0.44
5Ni/P25	54	0.02	0.08	0.38
P25-r	63	0.02	0.06	0.52
1Cu/P25-r	56	0.02	0.07	0.44
1Ni/P25-r	56	0.02	0.07	0.46
5Cu/P25-r	53	0.02	0.04	0.40
5Ni/P25-r	52	0.02	0.05	0.40

2.1.3. Transmission Electron Microscopy (TEM)

The particle size distribution of the metal species (Cu and Ni) deposited on TiO_2 was evaluated by TEM (Jeol JSM-IT500HR microscope, at 200 kV, and measured using the J-image software). A minimum number of 30 particles was selected for 1Cu/P25, 5Cu/P25, 5Cu/P25-r, and 5Ni/P25-r photocatalysts. For 1Cu/P25-r, only 14 particles were measured while for the rest of photocatalysts 1Ni/P25, 5Ni/P25, and 1NiP25-r, particles size measurement was not possible.

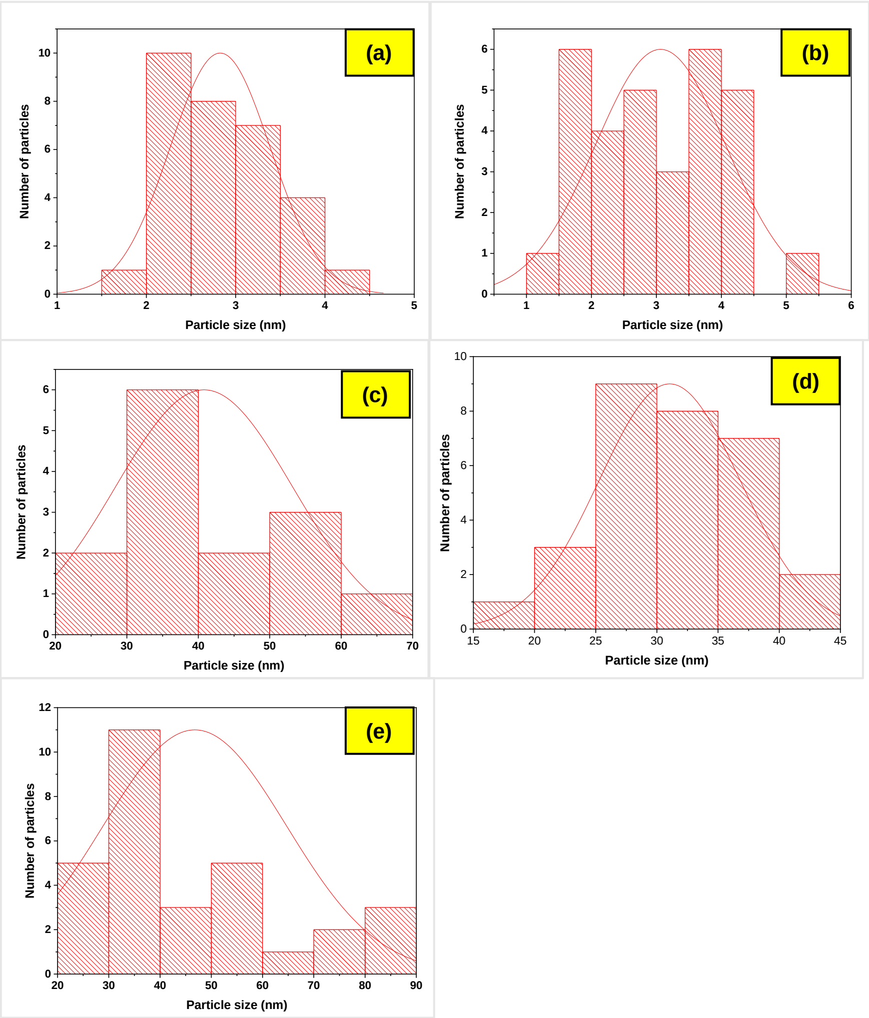

Figure S4. Particle size distribution for the metal particles supported on TiO_2 P25 in the different photocatalysts: a) 1Cu/P25, b) 5Cu/P25, c) 1Cu/P25-r, d) 5Cu/P25-r, and e) 5Ni/P25-r. (TEM analysis using Jeol JSM-IT500HR microscope, at 200 kV and the J-image software for particle size measurement). A minimum number of 30 particles except in the case of 1Cu/P25-r (only 14 particles) have been measured.

2.1.4. X-ray Photoelectron Spectroscopy (XPS)

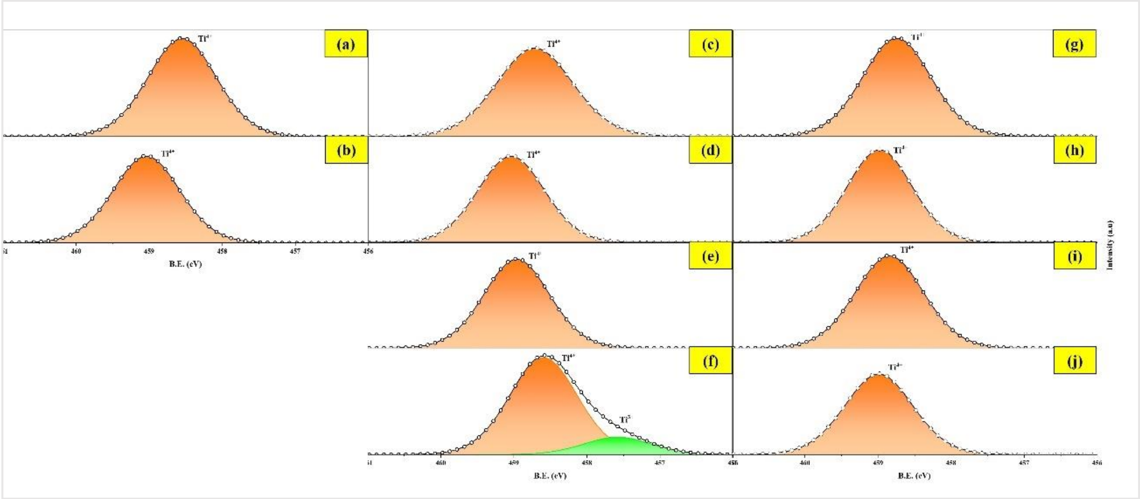

Figure S5. Ti 2p3/2 XPS spectrum for the studied photocatalysts: a) P25, b) P25-r, c) 1Cu/P25, d) 1Cu/P25-r, e) 5Cu/P25, f) 5Cu/P25-r, g) 1Ni/P25, h) 1Ni/P25-r, i) 5Ni/P25, j) 5Ni/P25-r. Measurements using a Thermo-Scientific NEXSA G2 instrument with Al Kα X-ray radiation (1486.6 eV) under ultra-high-vacuum conditions. Key parameters included a pass energy of 50 eV, a scan step of 0.1 eV, and sample irradiation within an ellipsoidal area with a major axis of 400 μm. The binding energies were calibrated using the C1s transition at 284.6 eV.

Table S2. XPS data of Ti 2p3/2 for all the studied photocatalysts.

Photocatalyst	Binding energy (eV)	Oxidation state	Proportion (%)
**P25**	458.55	Ti^{4+}	100
**1Cu/P25**	458.71	Ti^{4+}	100
**5Cu/P25**	458.96	Ti^{4+}	100
**1Ni/P25**	458.75	Ti^{4+}	100
**5Ni/P25**	458.85	Ti^{4+}	100
**P25-r**	459.04	Ti^{4+}	100
**1Cu/P25-r**	458.36	Ti^{4+}	100
**5Cu/P25-r**	457.58	Ti^{3+}	15
	458.59	Ti^{4+}	85
**1Ni/P25-r**	458.98	Ti^{4+}	100
**5Ni/P25-r**	458.99	Ti^{4+}	100

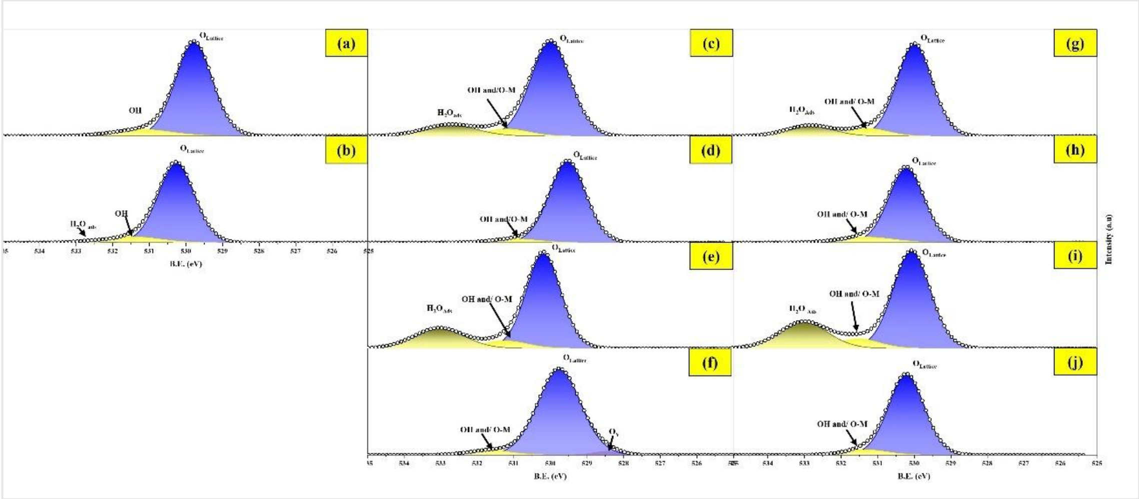

Figure S6. O 1s XPS spectrum of the studied photocatalysts: a) P25, b) P25-r, c) 1Cu/P25, d) 1Cu/P25-r, e) 5Cu/P25, f) 5Cu/P25-r, g) 1Ni/P25, h) 1Ni/P25-r, i) 5Ni/P25, j) 5Ni/P25-r. Measurements using a Thermo-Scientific NEXSA G2 instrument with Al Kα X-ray radiation (1486.6 eV) under ultra-high-vacuum conditions. Key parameters included a pass energy of 50 eV, a scan step of 0.1 eV, and sample irradiation within an ellipsoidal area with a major axis of 400 μm. The binding energies were calibrated using the C1s transition at 284.6 eV.

Table S3. XPS data of O 1s for all the studied photocatalysts.

Photocatalyst	Binding energy (eV)	Proportion (%)
**P25**	529.78	90
	531.19	10
**1Cu/P25**	530.00	80
	531.18	7
	532.76	13
**5Cu/P25**	530.19	71
	531.24	7
	533.05	22
**1Ni/P25**	529.99	81
	531.26	7
	532.87	12
**5Ni/P25**	530.07	68
	531.53	8
	533.03	24
**P25-r**	530.26	90
	531.39	9
	532.66	1
**1Cu/P25-r**	529.53	95
	530.97	5
**5Cu/P25-r**	528.46	3
	529.76	92
	531.68	5
**1Ni/P25-r**	530.21	90
	531.30	10
**5Ni/P25-r**	530.21	93
	531.33	7

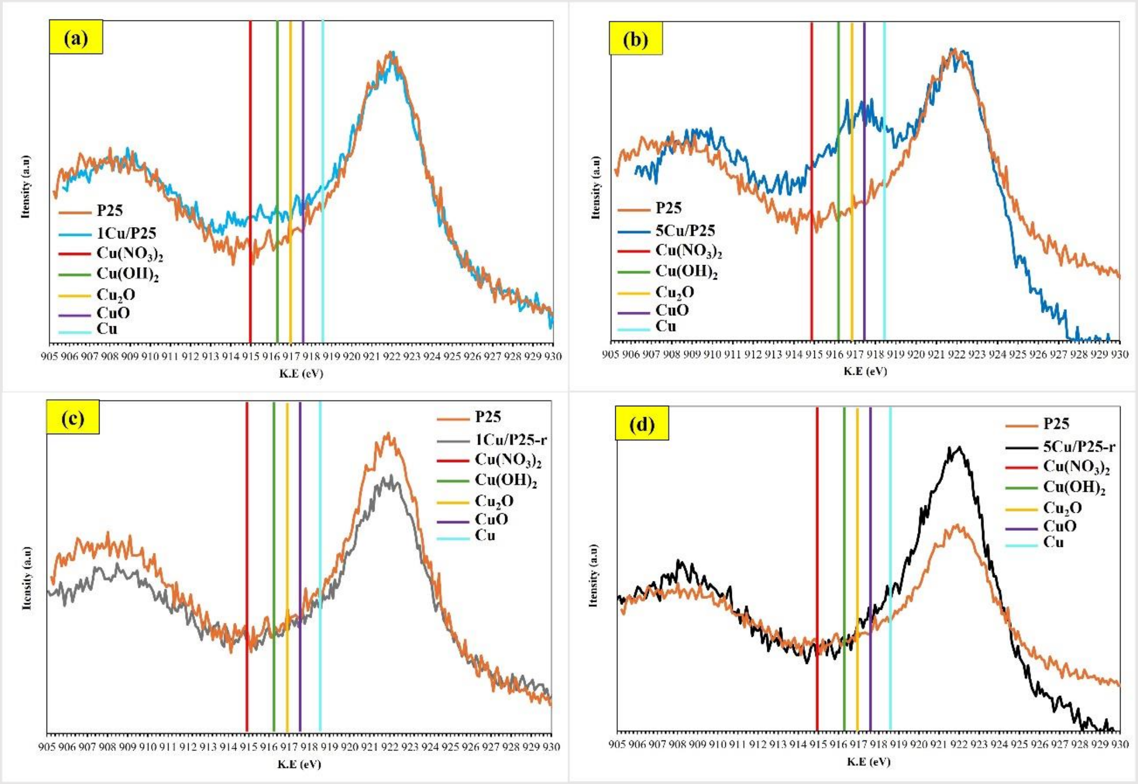

Figure S7. Auger spectra for: **a**) 1Cu/P25, **b**) 5Cu/P25, **c**) 1Cu/P25-r, and **d**) 5Cu/P25-r. Data for P25 are included in orange colour in each figure for comparison purposes.

2.1.5. Thermogavimetric (TG) analysis

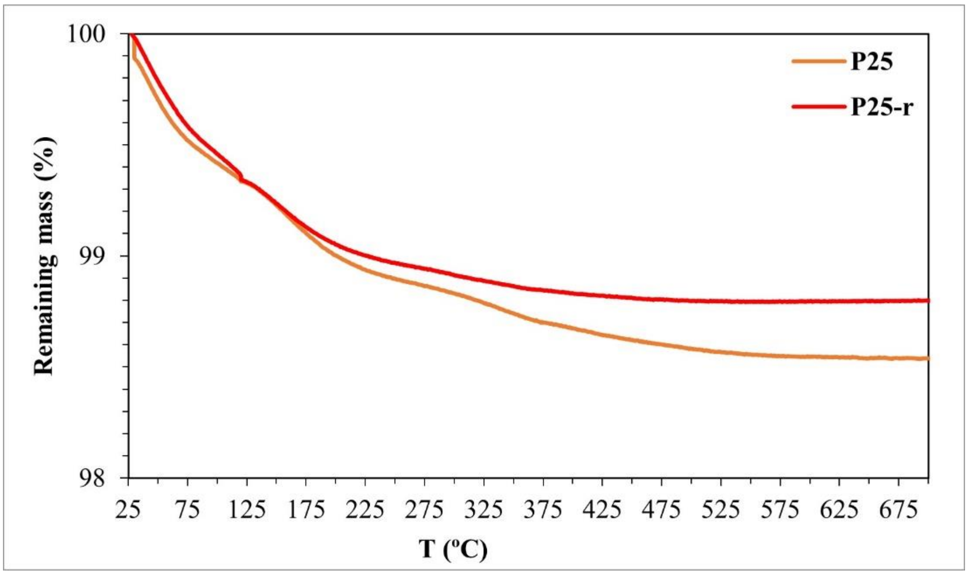

Figure S8. TG analysis of P25 and P25-r photocatalysts. Measurements were conducted in a SDT Q600 thermobalance (from TA Instruments), using about 10 mg sample The analysis conditions included a N_2 flow rate of 100 mL/min and a heating rate of 10 °C/min, following a three-stage temperature program: initial heating at 10 °C/min to 120 °C with 15 min isothermal period, followed by continuous heating at 10 °C/min up to 750 °C.

Table S4. Humidity and OH type-groups content calculated from TG analysis of P25 and P25-r photocatalysts.

Photocatalyst	Humidity (%)	OH_{weak} (%)	OH_{strong} (%)	OH_{total} (%)
**P25**	0.65	0.50	0.28	0.78
**P25-r**	0.62	0.45	0.12	0.57

2.1.6. Photoluminescence (PL)

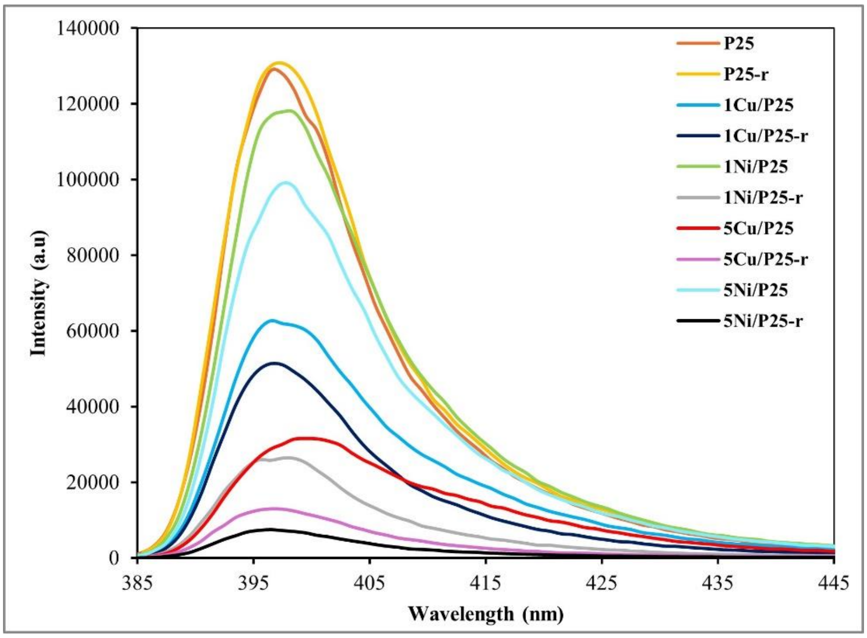

Figure S9. Photoluminescence spectra of the studied photocatalysts (before and after thermal reduction treatment).

2.2. UV-Photocatalyzed water splitting tests

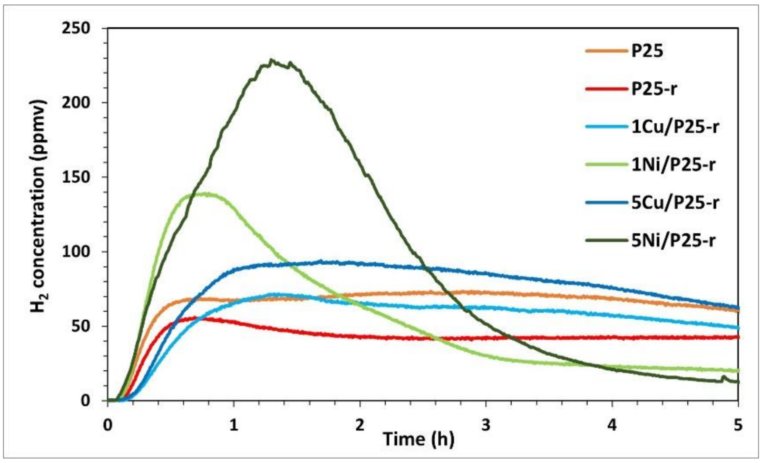

Figure S10. Hydrogen evolution vs time in the photocayzed water splitting tests performed using reduced metal titania photocatalysts (XM/P25-r). The water splitting tests using P25 or P25-r are also included for comparison purposes. The tests with XM-P25, for which hydrogen generation is negligible, have been omitted. The experimental conditions are: 20 mg of catalyst, 500 ml of distilled water, 30 ml/min He flow, stirring, 5h of irradiation.

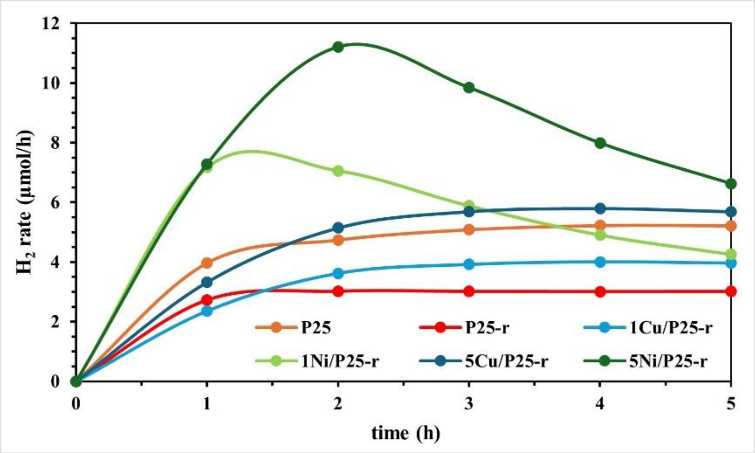

Figure S11. Hydrogen evolution rate (µmol/h) vs time in the photocayzed water splitting tests performed using reduced metal titania photocatalysts (XM/P25-r). The water splitting tests using P25 or P25-r are also included for comparison purposes. The tests with XM-P25, for which hydrogen generation is negligible, have been omitted. The experimental conditions are: 20 mg of catalyst, 500 ml of distilled water, 30 ml/min He flow, stirring, 5h of irradiation.

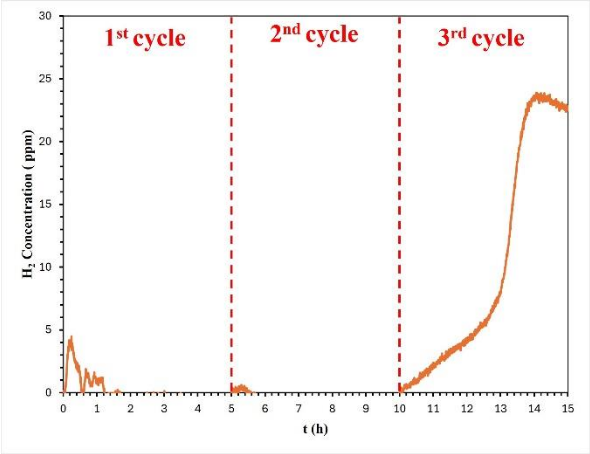

Figure S12. Hydrogen evolution from WS using 5Cu/P25 as a photocatalyst and 15 h of UV irradiation (3 irradiation cycles of 5 hours each, separated by 19 h in dark). The experimental conditions are: 20 mg of catalyst, 500 ml of distilled water, 30 ml/min He flow, stirring, 5h of irradiation per cycle, and 19h in darkness (in the inert atmosphere and under stirring) between each irradiation cycle.

Table S5. Hydrogen (H_2) generation over 5Cu/P25 during three successive irradiation cycles (5 hours each)

Cycle	H_2	
	µmol	$\mu mol \cdot g_{cat}^{-1}\ h^{-1}$
1	0.1	1
2	0.0	0
3	4.1	41

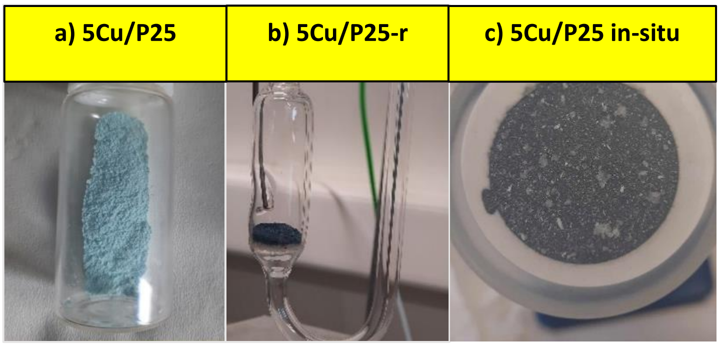

Figure S13. Comparison between Cu-containing photocatalysts: a) as prepared 5Cu/P25, b) thermally reduced 5Cu/P25-r, and c) 5Cu/P25 in-situ reduced during the catalytic test.

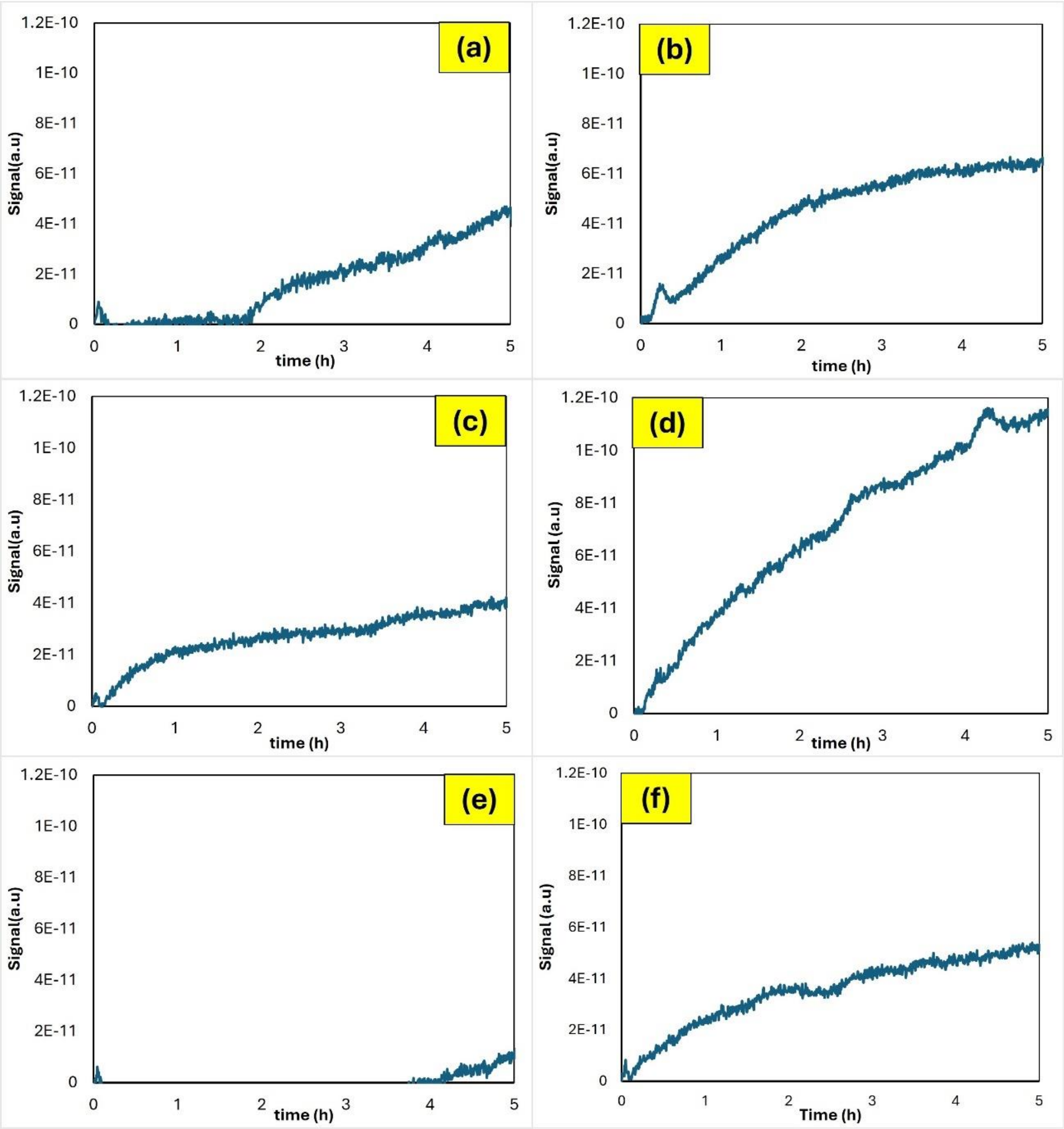

Figure S14. Signal (in a.u) of O_2 gas evolution during water splitting tests using: a) P25, b) P25-r, c) 1Cu/P25-r, d) 5Cu/P25-r, e) 1Ni/P25-r, and f) 5Ni/P25-r.

Table S6. Literature data compiling the conditions and amount of hydrogen generated by water splitting in photocatalysed processes (comparative data).

Photocatalyst	Light source	Peak wavelength (nm)	Power Or irradiance	H_2 amount		Ref
				µmol	µmol g_{cat}^{-1} h^{-1}	
TiO_2 anatase	UV (HeCd laser, 325 nm)	325	55 mW.cm^{-2}	-	0	[1]
Grey anatase					0	
1%Ni@grey anatase					50	
2%Ni@grey anatase					220	
3%Ni@grey anatase					700	
4%Ni@grey anatase					400	
5% Ni@grey anatase					100	
(1%) Ni/NiO-TiO_2 core shell	450 W xenon lamp		50 mW/cm^{-2}	3.25	-	[2]
Cu SA/BTO	simulated solar light		100 mW/cm^{-2}		881	[3]
Co SA/BTO					336	
Cu–Co SA/BTO					1238	
TiO_2/Co_3O_4/Ni(5% Co_3O_4/0.5% Ni)	UV-visible	-	-		123	[4]
TiO_2	Solar and simulated sunlight	λ max not mentioned	130000 Lux average solar sunlight intensity		55 (solar) and 29 (simulated	[5]
5.0Ni/TiO_2					160 (solar) and 154 (simulated	
2.0Cu/TiO_2					164 (solar) and 161 (simulated	
0.5Cu-5.0Ni/TiO_2					190 (solar) and 173 (simulated	
1.0Cu-5.0Ni/TiO_2					195 (solar) and173 (simulated	
2.0Cu-5.0Ni/TiO_2					198 (solar) and178 (simulated	
3.0Cu-5.0Ni/TiO_2					192 (solar) and 171 (simulated	

Pt/TiO_2 (Pt: 1%)	Xe lamp	320-780 nm	(50 W)	1.696	4	[6]
TiO_2	UV lamp Pen-Ray		4.4 MW/cm^{-2}	-	206	[7]
1.5 Ag/TiO_2					470	
P25	Hg UV lamp	365 nm	47 W	26.1	261	This work
P25-r				15.1	151	
1Cu/P25-r				19.8	198	
1Ni/P25-r				21.3	213	
5Cu/P25-r				28.4	284	
5Ni/P25-r				33.1	331	

In the work by N. Liu et al, Ni species were added to titania catalyst (commercial anatase) through a simple impregnation method followed by thermal reduction in H_2 atmosphere. Under these optimal conditions (3 wt.% of nickel loaded, and 500 ºC for the reduction in H_2, catalyst denoted as 3%Ni@grey anatase TiO_2), 700 µmol g_{cat}^{-1} h^{-1} were obtained. Notably, the use of anatase (metal-free) yielded no hydrogen, even after reduction. In addition, any Ni-loaded anatase catalyst without further thermal reduction shown H_2 formation (see Table S6, ref [1]).

In the study by Liuxian Zhang et al., the photocorrosion of Ni/NiO core/shell structures on TiO_2 in liquid water was investigated. A 1 wt% NiO/TiO_2 precursor was prepared via dry impregnation using $Ni(NO_3)_2 \cdot 6H_2O$ and anatase, followed by processing to create three samples: Ni/NiO-TiO_2 (calcined at 450 °C, reduced in 5% H_2/Ar at 500 °C, and partially oxidized in 100 Torr O_2 at 200 °C), Ni-TiO_2 (fully reduced at 500 °C without reoxidation), and NiO-TiO_2 (fully oxidized in air at 500 °C). Hydrogen generation was evaluated under a 450 W xenon lamp. Among the samples, only Ni/NiO-TiO_2 exhibited measurable H_2 production, generating approximately 3.25 µmol over 4 hours, with a maximum rate of ~5.5 g_{cat}^{-1} h^{-1} achieved within the first 100 minutes. However, the H_2 production rate declined over time, decreasing to half its initial value after 7 hours due to system deactivation. This deactivation was attributed to the photocorrosion of Ni metal, resulting in the loss of the Ni core, as confirmed by ICP and microscopy analyses. The absence of detectable oxygen, coupled with these observations, indicates that H_2 evolution occurred through a photochemical reaction between Ni and H_2O. Crucially, this process was not catalytic concerning Ni, as the Ni was consumed during the reaction, underscoring the central role of Ni photocorrosion in H_2 production (see Table S6, ref [2]).

Liang et al. further explored dual-single-atom Cu/Co dopants on Li-reduced blue TiO_2 (Cu–Co SA/BTO) under simulated solar irradiation. Cu SA/BTO achieved a hydrogen generation rate of 881 µmol g_{cat}^{-1} h^{-1}, which was 7.4 times higher than that of bare BTO. Co SA/BTO produced 336 µmol g_{cat}^{-1} h^{-1} and the Cu–Co SA/BTO catalyst exhibited an impressive hydrogen generation rate of 1238 µmol g_{cat}^{-1} h^{-1}. The improvement observed in the performance of metal-doped photocatalysts was attributed to the formation of O vacancies and the separation of photogenerated electrons and holes. Specifically, the introduction of Cu caused the transfer of electrons from the BTO conduction band to Cu atoms, while the Co-promoted the transfer of holes from the BTO valence band to Co atoms. (see Table S6, ref [3]).

Li et al. highlighted the potential of non-noble metal co-catalysts through their TiO_2/Co_3O_4/Ni catalyst (5% Co_3O_4/0.5% Ni). This photocatalyst was synthesized using a hetero-metal organic framework (H-MOF) template via a solvothermal approach, followed by photoreduction acheiving a hydrogen production rate of 123 µmol g_{cat}^{-1} h^{-1} under UV-visible light over three hours, an 8.7-fold improvement compared to TiO_2 (see Table S6, ref [4]).

This enhanced performance was attributed to the incorporation of Co_3O_4 as an oxidation cocatalyst and Ni as a reduction cocatalyst, which synergistically improved photocatalytic hydrogen evolution. Specifically, the porous Co_3O_4 structure enhanced optical absorption and promoted surface water oxidation kinetics, while in situ photo-deposited Ni facilitated electron transport and improved charge utilization efficiency.

In a study by Kumar et al., bimetallic (Cu-Ni) titania catalysts were investigated for hydrogen generation from water splitting. Catalysts were synthesized via co-impregnation and calcined at 450 °C for 5h. Hydrogen production was evaluated for various light sources (natural sunlight, artificial sunlight, visible light) with and without a sacrificial agent (methanol). The findings revealed superior catalytic activity for bimetallic catalysts compared to their monometallic counterparts. Interestingly, monometallic catalysts also outperformed bare TiO_2 catalysts. In tests without a sacrificial agent, the 2.0Cu-5.0Ni/TiO_2 catalyst exhibited the highest hydrogen formation, yielding 198 and 178 µmol g_{cat}^{-1} h^{-1} under solar and simulated sunlight, respectively (see Table S6, ref [5]). The investigation work by Yunlong Zhou et al. (ref [6]), principally dealt with studying the potential of a native lignocellulose biomass model, corn stover, as a sacrificial agent for enhancing hydrogen production from water splitting has used titania catalyst doped with platinum Pt (1 wt.% of Pt was incorporated to commercial titania Degussa P25 via photodeposition). For four hours of reaction time under UV-Visible illumination, the amount of hydrogen generated from water splitting was low (4 µmol. g_{cat}^{-1} h^{-1}).

Gogoi et al. (see Table S6, ref [7]) examined the impact of adding silver (Ag) to a TiO_2 catalyst for photocatalytic hydrogen production through water splitting, with and without sacrificial agents (alcohols and sulfur). They synthesized the Ag titania catalyst using a cost-effective chemical reduction method with ascorbic acid. Results indicated enhanced TiO_2 catalyst activity and hydrogen generation with 1.5 wt.% Ag incorporation, both with and without sacrificial agent presence. In case of no presence of a sacrificial agent, the hydrogen production rate was 470 µmol. g_{cat}^{-1} h^{-1} under the experimental conditions used.

References

[1] Liu, N.; Mohajernia, S.; Nguyen, N.T.; Hejazi, S.; Plass, Khant, A.; Yokosawa, T.; Osvet, A.; Spiecker, E.; Guldi, D.M. Guldi, Schmuki, P. Long-Living Holes in Grey Anatase TiO2 Enable Noble-Metal-Free and Sacrificial-Agent-Free Water Splitting Chem. Sus. Chem. 2020, 13, 4937–4944.

[2] Zhang, L.; Liu, Q.; Aoki, T.; Crozier, P.A. Structural evolution during photocorrosion of Ni/NiO Core/shell cocatalyst on TiO2 J. Phys. Chem. C 2015, 119, 7207–7214.

[3] Liang, M.; Shao, X.; Cho, Y.; Jadhav, A.R.; Hwang, Y.; Lee, J.; Kim, M.G.; Hong, Y.; Ajmal, S.; Yee, D.Y.; Tran, T.T.; Kim, J.; Bui, V.Q.; Ho, T.H.; Zhao, S.; Kim, Y.D.; Kim, J.H.; Lee, H. Effective Charge Separation in a Dual-Single-Atom Photocatalyst for Sacrificial Agent-Free H2 Evolution, ACS Sustain. Chem. Eng. 2024, 12, 6122–6131.

[4] Li, T.; Cui, J.D.; Xu, M.L.; Li, R.; Gao, L.M; Zhu, P.L.; Xie, H.Q.; Li, K. Engineering a hetero-MOF-derived TiO2-Co3O4 heterojunction decorated with nickel nanoparticles for enhanced photocatalytic activity even in pure water Cryst. Eng. Comm. 2020, 22, 5620–5627.

[5] Kumar, M.K.; Naresh, G.; Vijay Kumar, V.; Sasikumar, B.; Venugopal, A. Improved H2 yields over Cu-Ni-TiO2 under solar light irradiation: Behaviour of alloy nanoparticles on photocatalytic H2O splitting Appl. Catal. B 2021, 299, 120654.

[6] Zhou, Y.; Ye, X.; Lin, D. Enhanced photocatalytic hydrogen evolution by using alkaline pretreated corn stover as a sacrificial agent, Int J Energy Res 2020, 44, 4616–4628.

[7] Gogoi, D.; Namdeo, A.; Golder, A.K.; Peela, N.R. Ag-doped TiO2 photocatalysts with effective charge transfer for highly efficient hydrogen production through water splitting, Int J Hydrogen Energy 2020, 45, 2729–2744.
